# SUMOylation modification of HNRNPK at the K422 site promotes invasion in glioblastoma

**DOI:** 10.7150/ijbs.102051

**Published:** 2024-10-21

**Authors:** Wenguo Zhao, Jiazheng Wang, Feihu Zhao, Yaquan Li, Zhuo Li, Xingang Li, Anjing Chen

**Affiliations:** 1 Department of Neurosurgery, Qilu Hospital, Cheeloo College of Medicine and Institute of Brain and Brain-Inspired Science, Shandong University, Jinan, 250012, China.; 2 Jinan Microecological Biomedicine Shandong Laboratory and Shandong Key Laboratory of Brain Health and Function Remodeling, Jinan, 250017, China.

**Keywords:** HNRNPK, SUMOylation, glioblastoma stem cell, infiltration, proneural

## Abstract

Glioblastoma multiforme (GBM) is a highly heterogeneous brain tumor with limited treatment options. Recent studies revealed cellular heterogeneity and the potential for interconversion between distinct cell types on the basis of RNA sequencing and single-cell analyses. The ability of different cell types to adapt to their surrounding environment and undergo transformation significantly complicates the study and treatment of GBM. In this study, we reveal that HNRNPK-SUMO1 expression is predominantly found in the GBM infiltration area. SUMOylation of the K422 residue of HNRNPK interferes with its DNA binding ability, thereby disrupting downstream transcription, and ultimately leading to transitions between different states of glioblastoma stem cells. Although the proneural subtype is considered to have a better prognosis, transitioning towards this state promotes tumor invasion. These findings serve as a reminder to exercise caution when considering treatments targeting specific cellular subtypes.

## Introduction

Glioblastoma multiforme (GBM) is the most common primary malignant tumor in the brain. The new guidelines define GBM as glioma without isocitrate dehydrogenase (IDH) mutant 1. Multiple studies have described the characteristics of different tumor cells in glioblastoma. Verhaak's study, based on bulk RNA-seq, classified glioblastoma into proneural (PN), mesenchymal (MES), and classical (CL) subtypes 2, 3. Further single-cell-based studies have classified tumor cells into astrocyte-like (AC-like), mesenchymal-like (MES-like), neural progenitor-like (NPC-like), and oligodendrocyte progenitor-like (OPC-like) types 4. Soniya Bastola collected core tumor tissue (located within the T1 enhancement region) and peripheral tumor tissue (located outside the T1 enhancement region, but within the Fluid Attenuated Inversion Recovery (FLAIR) area) and described their characteristics 5. GBM is believed to be driven by a small population of glioblastoma stem cells (GSCs). GSCs can not only self-renew and differentiate into non-stem tumor cells but can also adapt to the microenvironment and transition into different states to drive tumor progression and recurrence. However, how this transformation is regulated internally within the cells remains unclear. Studying the characteristics of GSCs will help us gain a deeper understanding of the mechanisms underlying the development and progression of GBM and ultimately lead to better treatments for this disease.

Small Ubiquitin-like Modifier (SUMO)ylation is a protein modification process that involves the covalent attachment of SUMO proteins to target proteins following several enzymatic steps 6. First, the SUMO precursor protein is cleaved into its mature form by SUMO-specific proteases (SENPs). Next, the activating enzyme (E1) activates SUMO and forms a thioester bond with it. The activated SUMO is then transferred to the conjugating enzyme (E2). UBE2I, which is also named UBC9, is the only known SUMO E2. Finally, with the help of the SUMO ligase (E3), SUMO proteins are bound to their target proteins. E3 ligase is not required for SUMO modification, but its presence greatly enhances the efficiency of SUMOylation 7. SUMO1 molecules are covalently attached to specific lysine residues on target proteins under the mediation of E3 ligases. Several families of E3 ligases have been identified, including the RanBP2, TRIM, and PIAS families 7, 8. Different cells and proteins often have different E3 ligases for SUMOylation. The dynamics of SUMOylation allow proteins to be rapidly and reversibly modified, enabling the fine-tuning of cellular processes in response to various stimuli and environmental changes. SUMOylation primarily targets nuclear proteins and affects transcriptional regulation, DNA repair, and chromatin structure 7. Therefore, understanding the mechanisms and functional consequences of SUMOylation is crucial for elucidating cellular processes and developing potential therapeutic strategies.

In this study, we investigated the expression of SUMO-modified related molecules in glioblastoma and the role of HNRNPK in SUMO modification. Molecules associated with SUMOylation were found to be highly expressed in areas of tumor microvascular proliferation and showed a preference for expression in NPC-like tumor cells. According to our research, environmental factors such as hypoxia can regulate intracellular SUMOylation. The SUMOylation of lysine at the K422 site on the third KH domain of HNRNPK can disrupt its binding to DNA, thereby interfering with transcriptional regulation and altering GBM cell states. The transition towards the proneural/infiltration subtype improves survival, but it also leads to increased proliferation of tumor cells in the edge and infiltration of tumor cells into brain tissue.

## Material and methods

### Experimental model and study participant details

The protocol for this study (DWLL-2021-109) was approved by the Ethics Committee of Qilu Hospital, Shandong University. All relevant laws, regulations and guidelines were followed in this study. Human GBM tissue samples were obtained from patients undergoing surgery at Qilu Hospital. Written informed consent was obtained from all patients prior to participation. GBM stem cells (P3#GSC, BG5#GSC, and BG7#GSC) were previously isolated and characterized 39.

### Cell culture and treatment

GSCs were cultured in neurobasal medium (A2477501, Gibco/Thermo Fisher Scientific) supplemented with 2% B-27 Neural Mixture (17504044, Gibco/Thermo Fisher Scientific), 10 ng/mL EGF, and 10 ng/mL bFGF. Cell differentiation was induced by treating the cells with 10% FBS for 48 h. The low-oxygen treatment (CO_2_ 5%, O_2_ 1%, 37℃) involved culturing the cells in a hypoxic incubator for 48 h, while the UV treatment (254nm) consisted of exposing the cells to UV radiation for 8 h.

### Immunofluorescence

We collected tissue samples from five GBM patients (IDH wt). Sodium citrate antigen retrieval solution (C1032, Solarbio) was added to the slides. The slides were heated on low heat for 20 min and then cooled in an ice water bath to room temperature. The primary antibody was incubated overnight at 4°C, followed by 30 min of re-warming at room temperature. HRP-conjugated secondary antibody was incubated at room temperature for 1 h, followed by rinsing with PBS. FITC-Tyramide (G1222-50UL, Servicebio) was incubated at room temperature for 10 min, followed by washing with TBST. The same method was applied to stain other molecules using CY3-Tyramide (G1223-50UL, Servicebio). The specific information of the antibody is provided in the Key resources table.

### Xenograft intracranial tumor assay

Four-week-old male BALB/c-nude mice (Jiangsu Jicuiyaokang Biotech Co., Ltd., China) were bred under SPF conditions. All mice (five per group) were anesthetized using isoflurane gas and fixed in a stereotaxic frame. The injection site was located 1 mm anterior and 2 mm to the right of the bregma, with an injection depth of 2.5 mm. Each mouse was injected with 3 × 10^5^ P3#GSCs or BG5#GSCs. The tumor volume was evaluated using a bioluminescence imaging system (IVIS Spectrum, PerkinElmer; Waltham, MA, USA). Euthanasia was performed when mice exhibited weight loss, loss of appetite, weakness, or were in a moribund state, or when they showed neurological or behavioral symptoms.

### Immunohistochemistry

The paraffin sections were deparaffinized using xylene, followed by antigen retrieval using EDTA antigen retrieval solution. The primary antibody was incubated overnight at 4°C. After re-warming to room temperature for 30 min, the secondary antibody was incubated for 1 h, followed by DAB staining. Details of the antibodies can be found in the Supplementary Table S2. Whole-slide images were imported into Qupath 40 (Version: 0.4.3) for data analysis. The specific information of the antibody is provided in the Key resources table.

### Surface plasmon resonance (SPR)

HNRNPK (WT), KH3 (WT), and KH3 (K36R) were cloned into the pet28a vector with C-terminal fusion of the HIS tag. The bacterial strains were amplified in LB medium, and protein expression was induced using isopropyl ß-D-1-thiogalactopyranoside (I6758, Merck). Proteins were purified using His-tag purification, the efficiency of which was verified using Coomassie brilliant blue or silver staining. For the SUMOylation modification of the protein, the KH3 (WT) protein was subjected to *in vitro* SUMOylation using the SUMOylation kit (BML-UW8955-0001, Enzo). Mix 2.0μl of SUMOylation buffer, 1μl of Mg-ATP, 1μl of 20x SUMO E1, 1μl of SUMO E2, 1μl of SUMO-1, and 1μM of the target protein on ice. Then incubate the mixture at 37ºC for 60 minutes. The effect of SUMO modification was detected using silver staining. The SADH (19-0130, Octet® SPR Sensor Chip) chip was used to bind biotinylated ssDNA (CTCAGCCTCCCGACTC), and the binding response units (RUs) between different proteins and ssDNA were measured using the SPR system. The results were processed using Qdat software to generate binding curves and analyze the binding constants.

### GBM-brain organoid co-culture invasion assay analysis

Mouse embryonic brain organotypic cells were cultured for 21 days as described 41. GBM cells expressing GFP were cultured in low-adhesion 96-well plates to generate glioma spheres and then co-cultured with mature brain organotypic slices for 48 h. GBM cell invasion images were captured using a confocal microscope (Leica TCS SP8; Wetzlar, Germany). See Supplementary information for additional details on data analysis.

### Quantification and statistical analysis

The Shapiro-Wilk test was used to assess the normality distribution of the data, whereas the Bartlett test was employed to evaluate the homogeneity of variances. For comparisons among multiple groups, either ANOVA or the Kruskal-Wallis test was performed to determine if there were significant differences. Either the t-test or Wilcoxon test was used to examine pairwise differences between groups. Post-hoc tests were performed using TukeyHSD or kwAllPairsNemenyiTest. All analyses were performed using R 4.2.3.

See Supplementary information for additional details on data analysis.

### Data and code availability

The sequencing data has been uploaded to the GEO database, with the accession number GSE262681. Any additional information required to reanalyze the data reported in this work paper is available from the lead contact upon request. This paper does not report original code.

## Results

### SUMO1 modification is primarily expressed in NPC-like GBM cells

**Figure 1:**
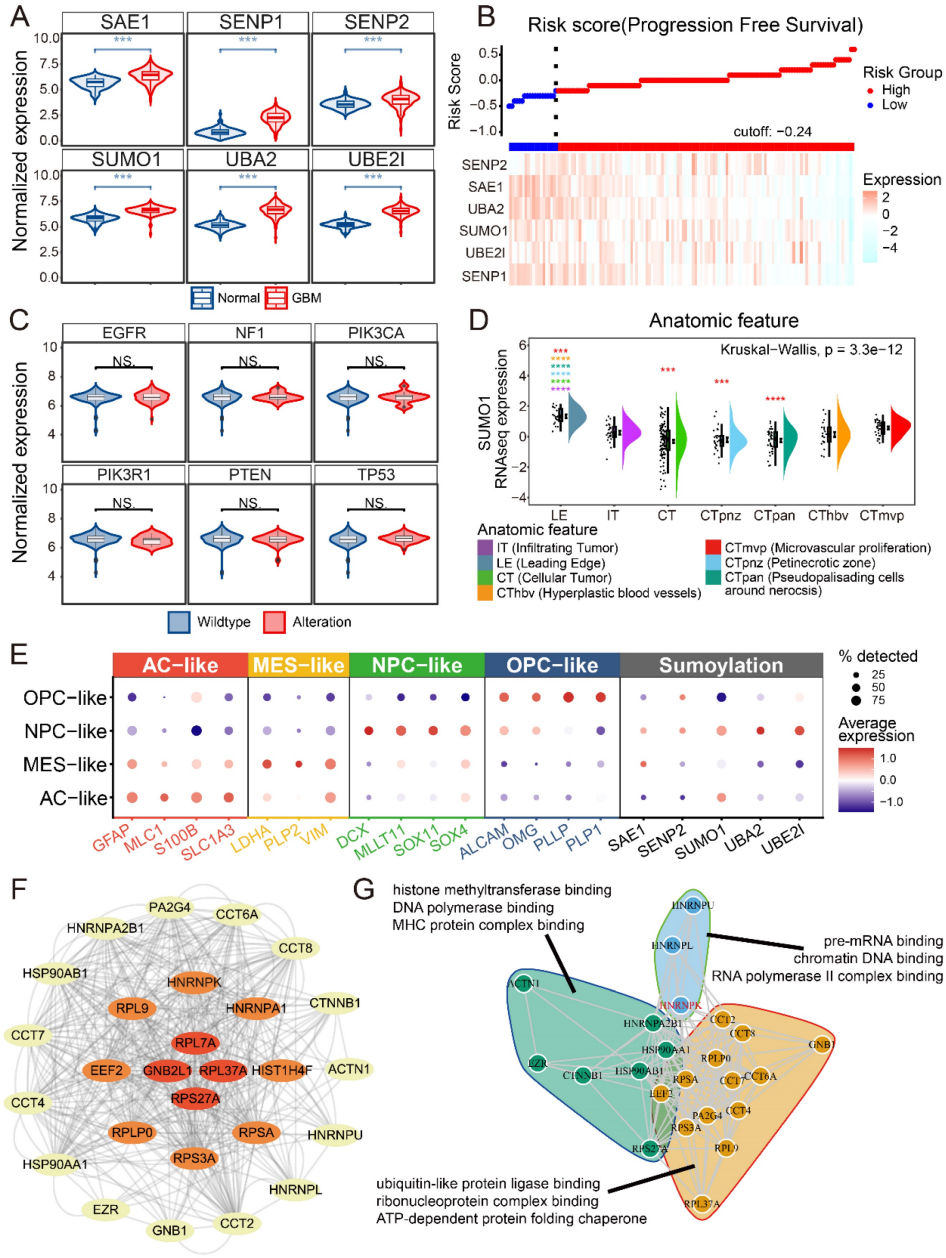
** Data analysis of SUMOylation-associated genes.** A. The expression of SUMO-related molecules in GBM and normal brains. B. Risk score of progression free survival and expression of SUMOylation-associated molecules. C. SUMO1 expression in wild-type or alteration sample of GBM. Alterations include homologous deletion, amplification, mutation, and fusion. D. SUMO1 expression in different anatomic tumor region. The data were obtained from IVY. E. Dotplot showing expression of different tumor cell markers and SUMOylation associated molecules in different single cell types. The single cell data were obtained from GSE159416. F. SUMOylated proteins interaction results from the STRING database. The images were generated using Cytoscape. G. Enrichment analysis and cluster of proteins SUMOylated. *p < 0.05, **p < 0.01, ***p < 0.001, ****p ≤ 0.0001.

First, we examined the expression of SUMO-modified related molecules in glioblastoma and normal brain tissue. The expression of several SUMOylation-related molecules was investigated and found to be highly expressed in GBM compared to normal brain tissue (Figure 1A). Risk score and survival analyses revealed that patients with high expression of SUMOylation-related molecules had improved progression-free survival (PFS) (Figure 1B and Supplementary Figure 1). The relationship between SUMO1 expression and common GBM mutations, as well as bulk-RNAseq scoring in GBM samples, was examined (Figure 1C and Supplementary Figure 2) 9, 10. No significant relationships were observed between SUMO1 expression and mutations (Figure 1C). We found no significant correlation (|R| < 0.3) between SUMO1 expression and GBM scoring. Querying the IVY database based on sequencing results from anatomical locations revealed that SUMO1 was predominantly expressed at the leading edge and microvascular proliferation areas (Figure 1D) 11. To analyze the cellular distribution of SUMO modification in GBM, single-cell data were downloaded and subjected to dimensionality reduction and annotation based on current research 4, 12, 13. The results indicated high expression of SUMOylation-related molecules in NPC-like GBM cells (Figure 1E), which are considered to be the main invasive cells 14. The results suggest that SUMO modification primarily occurs in NPC-like cells located at the tumor periphery and around blood vessels.

Our previous study examined proteins SUMOylated in GBM (Supplementary Table S1). The study employed Anti-K-ε-GG antibody beads to extract proteins SUMOylated, followed by positive ion detection using a Q-Exactive mass spectrometer. The raw sample data were processed and searched using MaxQuant software for data consolidation and analysis. The protein-protein interaction network of SUMOylated proteins in GBM was analyzed using the STRING database (Figure 1F) 15, 16. Functional enrichment analysis and clustering of the proteins suggested that SUMO1-modified proteins primarily regulate protein-protein interactions and are associated with DNA-RNA binding and editing (Figure 1G). Due to the central role of HNRNPK in both protein interactions and functional clustering, and HNRNPK is primarily modified by SUMO1 17, we further investigated the SUMOylation function of HNRNPK in GBM.

### HNRNPK-SUMO1 is primarily localized in the GBM infiltrating region

**Figure 2:**
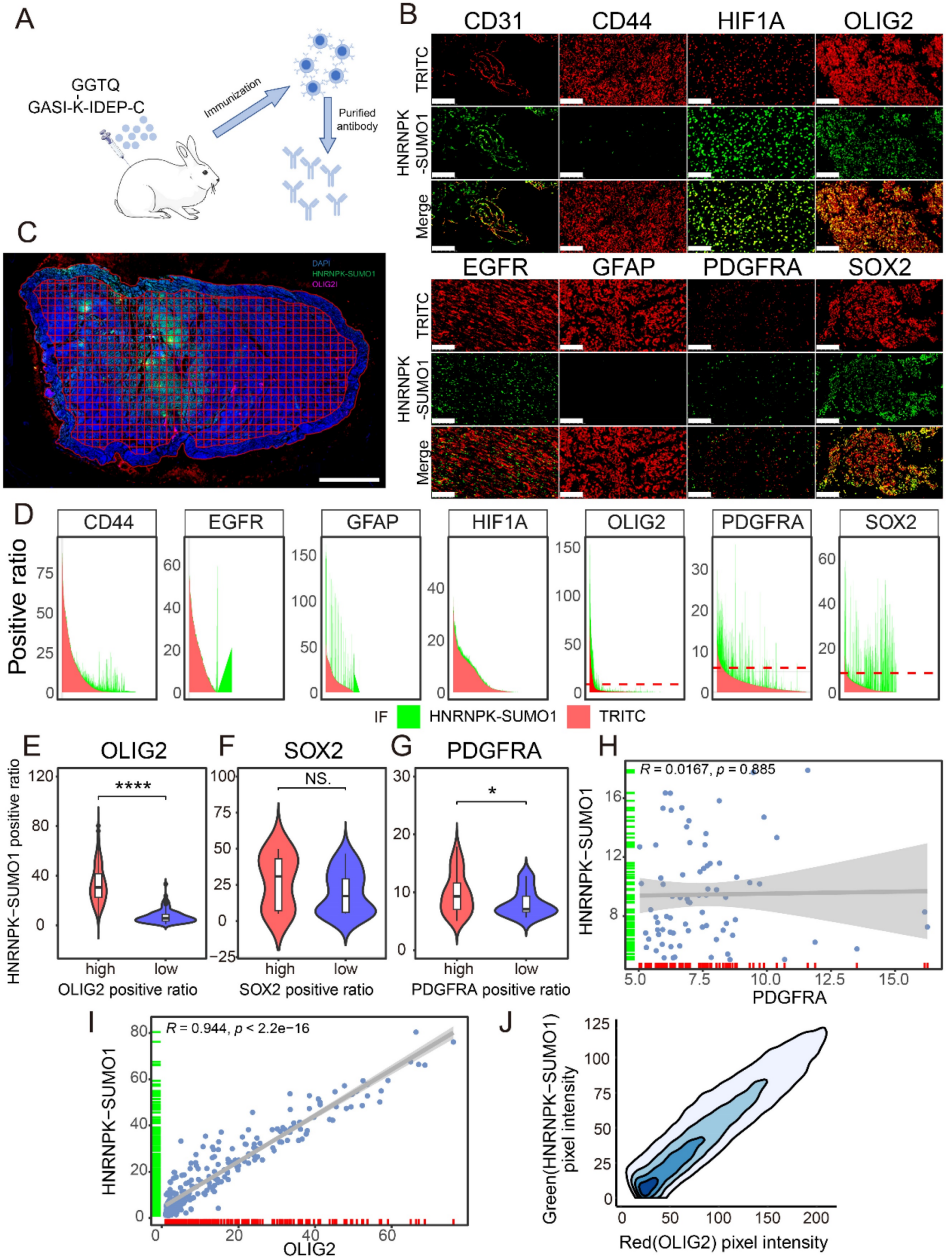
** Immunofluorescence distribution of HNRNPK-SUMO1.** A. Schematic diagram of HNRNPK-SUMO1 antibody generation. B. Immunofluorescence co-staining images of HNRNPK-SUMO1 with CD31, CD44, HIF1A, OLIG2, EGFR, GFAP, PDGFRα, and SOX2. Scale bars: 100 μm. C. Immunofluorescence image of the entire tissue output by Qupath. Scale bars: 2 mm. D. Area plot illustrates the positivity rates of red and green fluorescence in all tiles; the red color represents the positivity rate of the corresponding molecule in each title, while the green color represents the positivity rate of HNRNPK-SUMO1. E-G. Violin plots are used to display the differences in HNRNPK-SUMO1 positivity rates between high and low groups of OLIG2, SOX2, and PDGFRA. The minimum p-value between groups was calculated to set the threshold. H-I. Correlation analysis of HNRNPK-SUMO1 with the fluorescence positivity rates of OLIG2 or PDGFRA. J. Density plot of the fluorescence distribution of OLIG2 (red) and HNRNPK-SUMO1 (green). *p < 0.05, **p < 0.01, ***p < 0.001, ****p ≤ 0.0001.

Previous studies have confirmed the occurrence of SUMO modification of HNRNPK at residue 422 and HNRNPK is primarily modified by SUMO1, but the functional significance of this SUMO modification in GBM remain unclear 18. A modified peptide, GASI-(K-ε-GGTQ)-IDEP-C, was synthesized to create an antibody specifically recognizing the SUMO1 modification at the K422 site of HNRNPK (Figure 2A). The antibody was validated through co-immunoprecipitation experiments and western blotting (Supplementary Figure 3A-B). Immunofluorescence co-staining was performed to examine the localization of HNRNPK-SUMO1 with various cell markers (Figure 2B). We observed that several markers could detect varying degrees of colocalization with HNRNPK-SUMO1. To further quantify and clarify the distribution of HNRNPK-SUMO1, QuPath software was used to divide the tissue into tiles, and the positivity rates of different markers were calculated in each tile (Figure 2C). We quantified the fluorescence positivity rate of each marker in more than 2000 tiles from five GBM samples. We observed a high degree of overlap in the distribution of SOX2, OLIG2, and PDGFRA with HNRNPK-SUMO1 (Figure 2D). Differential analysis indicated that HNRNPK-SUMO1 showed high expression in regions where OLIG2 and PDGFRA were also highly expressed (Figure 2E-G), and correlation analysis confirmed a high degree of overlap in the positivity rates of OLIG2 and the distribution of HNRNPK-SUMO1 (Figure 2H-I). Fluorescence scatter analysis also demonstrated significant co-localization between OLIG2 and HNRNPK-SUMO1 (Figure 2J).

**Figure 3:**
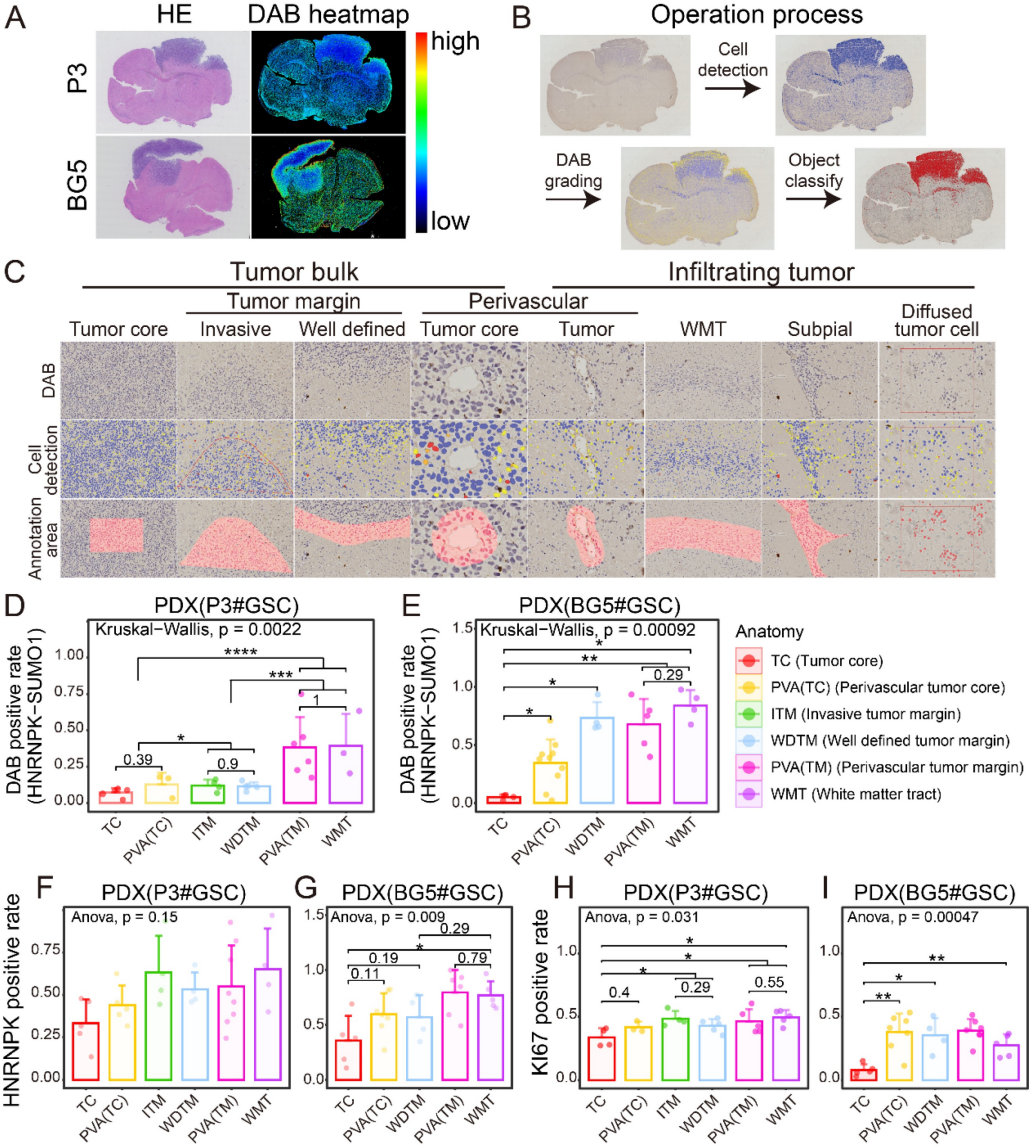
** Immunohistochemistry location of HNRNPK-SUMO1.** A. HE stain and HNRNPK-SUMO1 DAB stain of tissue sections. B. Flowchart illustrating the data processing pipeline. C. Cell detection and annotation of anatomic sections from PDX models. D-E. HNRNPK-SUMO1 positive rates in different anatomic regions of GBM. Data are presented as the mean ± SD. F-G. HNRNPK positive rates in different anatomic regions of GBM. Data are presented as the mean ± SD. H-I. Ki67 positive rates in different anatomic regions of GBM. Data are presented as the mean ± SD. *p < 0.05, **p < 0.01, ***p < 0.001, ****p ≤ 0.0001.

OLIG2 is considered a key molecule involved in mediating single-cell mode invasion of GBM cells and is highly expressed at the invasive tumor edge 19. To explore the tissue localization of HNRNPK-SUMO1, P3#GSC, and BG5#GSC were utilized to establish a patient-derived tumor xenograft (PDX) model. From the heatmap distribution of HNRNPK-SUMO1, we observed a higher distribution of HNRNPK-SUMO1 at the tumor periphery compared to the tumor core (Figure 3A). To further validate this, we performed quantitative analysis of tissue section staining in distinct regions. The tumor edge can be divided into the invasive margin or well-defined margin according to Alieva's research 20. The tumor invasion areas beyond the tumor bulk were classified as infiltration regions. Based on Scherer's classification of glioma invasion 21, we annotated the tumor invasion regions (Figure 3B-C). HNRNPK-SUMO1 was primarily present at the tumor infiltrating edges, while the positivity rate of HNRNPK-SUMO1 in the tumor core region was significantly lower (Figure 3D-E). However, we found no significant differences in the expression of HNRNPK in different regions (Figure 3F-G). Evaluation of cell proliferation (Ki67) also revealed a significantly higher proliferation rate at the tumor periphery than at the tumor core (Figure 3H-I).

### SUMO1 modification interferes with the binding ability of HNRNPK to ssDNA

**Figure 4:**
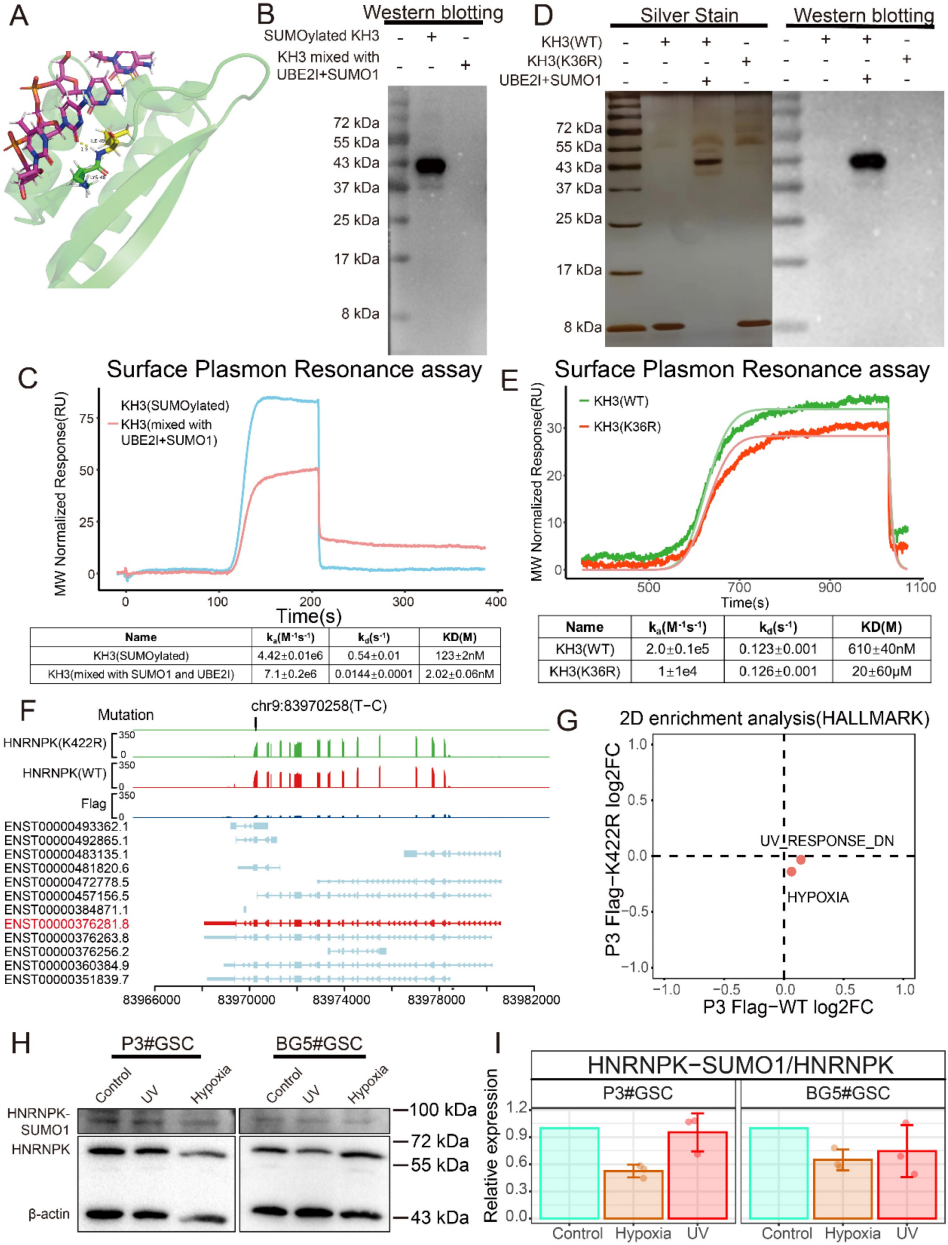
** SUMOylation of HNRNPK inhibits ssDNA binding affinity and is regulated by environmental factors.** A. Protein structure of KH3. The structure (1j5k) was acquired from the PDB database. B. Western blotting of SUMOylated KH3 and KH3 (mixed with UBE2I and SUMO1). C. ssDNA and KH3 or SUMOylated KH3 affinity binding curves. D. Protein silver stain and western blotting of KH3 (WT), KH3 (K422R), and SUMOylated KH3. E. ssDNA and KH3 (WT) or KH3 (K422R) affinity binding curves. F. TrackViewer displays all transcripts of HNRNPK, along with the results of RNA-seq and the mutation positions identified by GATK4. G. Two-dimensional enrichment analysis revealed differentially activated pathways between the overexpressed HNRNPK (WT) and HNRNPK (K422R) treatments in P3#GSC. H. Western blotting examining the expression of HNNRPK-SUMO1 in P3#GSC and BG5#GSC treated with UV or hypoxia. I. Western blotting quantitative results of HNNRPK-SUMO1 expression in P3#GSCs and BG5#GSCs treated with UV or hypoxia. Data are presented as the mean ± SD.

*HNRNPK* contains three KH domains, and the modification at position 422 is located on the third KH domain of this molecule. The KH3 domain adopts a β-α-α-β-β-α structure and can specifically recognize the TCCC sequence of ssDNA 22 (Figure 4A). Initially, attempts were made to construct full-length HNRNPK using the ENST00000376281.8 transcript. However, it was found that the transcripts of *HNRNPK* cannot all fold into the target size protein *in vitro*, making it difficult to purify the desired region (55-70 kDa) of the protein (Supplementary Figure 4A). Therefore, the KH3 domain of HNRNPK (387-451 aa) was constructed to explore the function of SUMO1 modification. The purified KH3 domain protein was subjected to SUMO1 modification, and the antibody recognizing the SUMOylation of the specific K422 site on HNRNPK was used for western blotting to confirm the *in vitro* modification (Figure 4B). Following SUMOylation, the KD value significantly increased, suggesting a decrease in affinity for KH3 binding ssDNA (Figure 4C). Given that it is not feasible to establish a stable SUMO1-modified state of the target protein in an *in vivo* environment, current studies on the function of SUMO1-modified proteins often involve fusing the SUMO protein to the N-terminus of the target protein or introducing specific site mutations to investigate the impact of SUMOylation on the protein. In this study, relevant proteins were constructed by mutating the lysine residue at position 36 (corresponding to HNRNPK's K422 site) on the KH3 domain, and the effect of the mutation on binding ability was analyzed. Mutation of K36 to R resulted in a significant decrease in the binding rate (k_a_) with ssDNA, leading to a decrease in its binding capacity (Figure 4D-E). We also found that the mutation suppresses the binding affinity of the KH3 domain of HNRNPK to ssDNA, and its impact on the function of the KH3 domain is consistent with SUMO1 modification.

In this study, wild-type (WT) and K422R mutant HNRNPK with GFP tags were constructed using the ENST00000376281.8 transcript to explore the function of HNRNPK and the impact of disrupting HNRNPK and DNA binding on GSCs (Figure 4F) 23. Immunofluorescence analysis revealed that both the WT and mutant forms were expressed in the nucleus and cytoplasm of cells, with a slight increase in nuclear localization observed for the K422R mutation and HNRNPK-SUMO1 than wild-type HNRNPK (Supplementary Figure 4B-C). To identify potential factors that may influence SUMO1 modification of HNRNPK, we performed functional enrichment analysis on overexpressed wild-type and mutant GSCs. Two-dimensional enrichment (2D-enrichment) analysis revealed differential activation of pathways in P3#GSCs in response to ultraviolet (UV) and hypoxic treatment (Figure 4G). Subsequently, the cells were exposed to UV radiation for 8 h or hypoxic treatment for 48 h *in vitro*. Western blotting showed that hypoxia treatments led to a decrease in the level of SUMOylation modification of HNRNPK (Figure 4H-I).

Protein co-immunoprecipitation experiments were conducted to detect proteins bound to HNRNPK. A significant decrease in the abundance of proteins bound to HNRNPK was observed when the K422 residue was mutated (Supplementary Figure 5A). Functional clustering analysis of proteins bound to HNRNPK showed enrichment in DNA and RNA binding. Specifically, HNRNPK (WT) exhibited more interactions with proteins associated with translation, RNA polymerase, and RNA helicase activity, compared to the mutant form (Supplementary Figure 5B-C).

### HNRNPK regulated the transition of the GSC state

**Figure 5:**
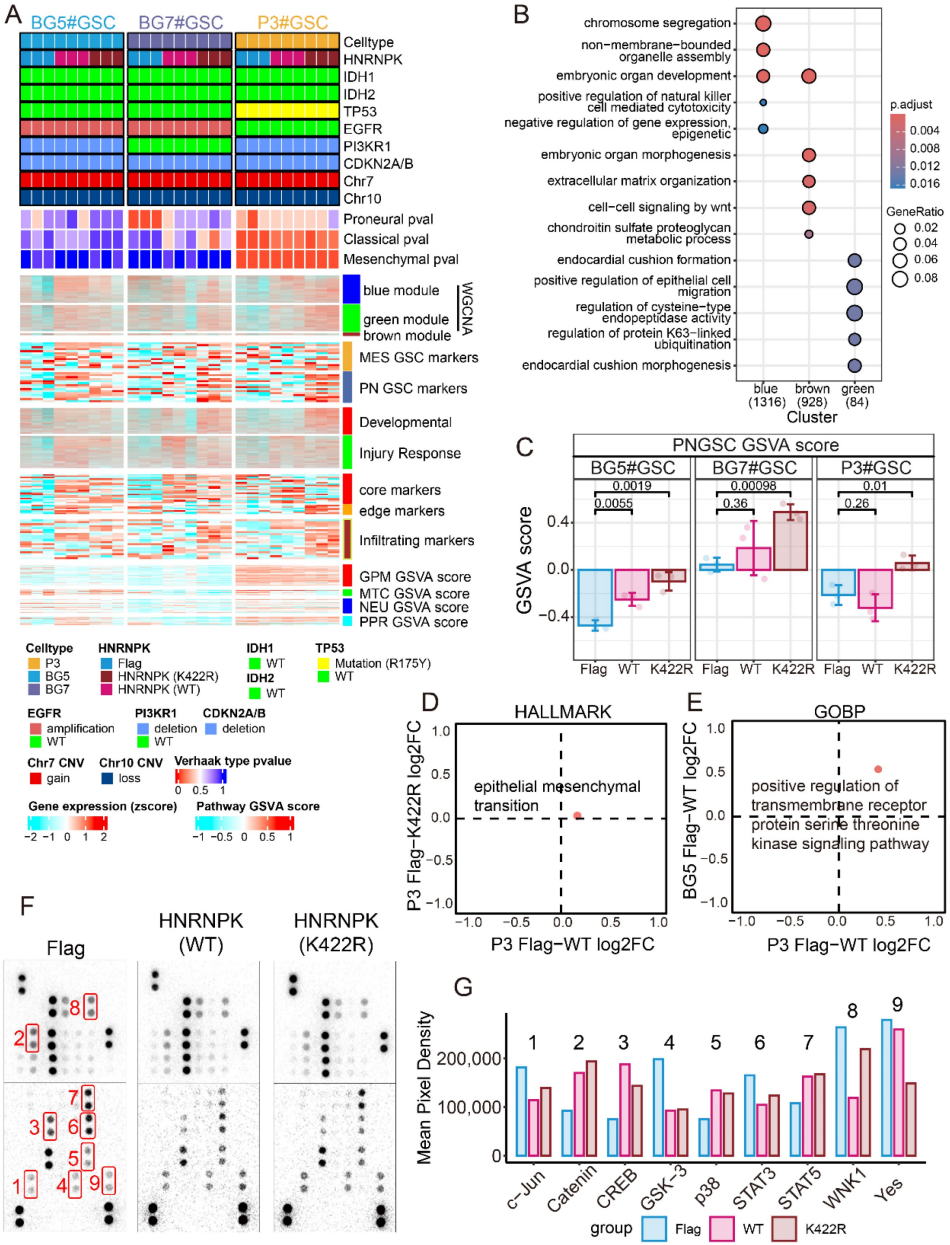
** HNRNPK regulates the subtype transition of GSCs.** A. Complex heatmap of GSC mutation status, subtype, WGCNA module, expression of different markers, and GSVA scores. The expression of markers was z-score normalized across different GSC groups. B. GO enrichment analysis of WGCNA module. C. Boxplot presenting the RNAseq GSVA scoring results of GSCs (P3, BG5 and BG7) overexpressing Flag, WT (HNRNPK), and K422R (HNRNPK). D. Two-dimensional enrichment analysis revealed differentially activated pathways between the overexpressed HNRNPK (WT) and HNRNPK (K422R) treatments in P3#GSC. E. Two-dimensional enrichment analysis revealed both activated pathways between the overexpressed HNRNPK (WT) in P3#GSC and BG5#GSC. F. Proteome Profiler Human Phospho-Kinase Array results of P3#GSC overexpressing Flag, HNRNPK (WT), or HNRNPK (K422R). G. Differential pixel intensity of the Proteome Profiler Human Phospho-Kinase Array assay.

To elucidate the function of HNRNPK, RNA-seq was performed in three types of GSCs overexpressing WT or mutant HNRNPK, namely P3#GSC, BG5#GSC, and BG7#GSC (Figure 5A) 24 25 26. WGCNA was used to identify key modules in different treatment groups (Supplementary Figure 6A). In our study, we found that WGCNA primarily enriched three distinct modules: one module associated with negative regulation of gene expression, and the remaining two modules related to extracellular matrix and cell migration (Figure 5B). Based on these findings, we hypothesize that HNRNPK is functionally involved in transcriptional regulation and cell invasion. The module-trait analysis in WGCNA further suggests a strong correlation between these modules and cellular subtype and tissue localization (Supplementary Figure 6B). So we investigated the expression changes of different markers and observed that GSCs overexpressing HNRNPK (K422R) significantly increased the expression of markers in the infiltrating region, whereas GSCs overexpressing HNRNPK (WT) tended to increase the expression of markers in the core region (Figure 5C and Supplementary Figure 7)^27^. We compared the differentially activated pathways between P3#GSCs overexpressing HNRNPK (WT) and HNRNPK (K422R). These two treatments exhibited differential activation efficiency in the epithelial-mesenchymal transition pathway (Figure 5D). Only HNRNPK (WT) can activate epithelial-mesenchymal transition pathway. Next, gene set enrichment analysis (GSEA) was conducted on bulk RNA-seq data. We found that the differences primarily lay in the E2F target, G2M checkpoint, RHO GTPase cycle, and KARS signaling pathways. This also suggests that HNRNPK perform function in transcription and the regulation of cell proliferation and invasion (Supplementary Figure 8).

Based on the 2D-enrichment results of P3#GSC and BG5#GSC, we identified the protein serine threonine kinase signaling pathway as being commonly activated upon overexpression of HNRNPK (WT) (Figure 5E). Due to the roles of HNRNPK in cellular phosphorylation, we further investigated the differences between treatments using a phosphorylation array (Figure 5F). As a result, we found differences in the phosphorylation levels of p-c-JUN, p38, and p-CREB (Figure 5G). Combining the results from the phosphorylation array and functional pathway enrichment analysis, we performed western blotting experiments to examine commonly activated phosphorylation signaling pathways in GBM. Compared to cells overexpressing HNRNPK (K422R), overexpression of HNRNPK (WT) upregulated proteins such as p-CREB, p38 and P65, which were highly expressed in the MES#GBM subtype (Supplementary Figure 9). By overexpressing SUMO1 and UBE2I, we enhanced the levels of intracellular SUMOylation modification. Although there was no statistically significant difference, overexpression of SUMO1, UBE2I and HNRNPK (WT) led to a decrease of p-CREB, p38 and P65 comparing to cells only overexpressing HNRNPK (WT) to varying degrees.

### SUMOylation of HNRNPK interferes with transcriptional inhibition of TSPAN13

**Figure 6:**
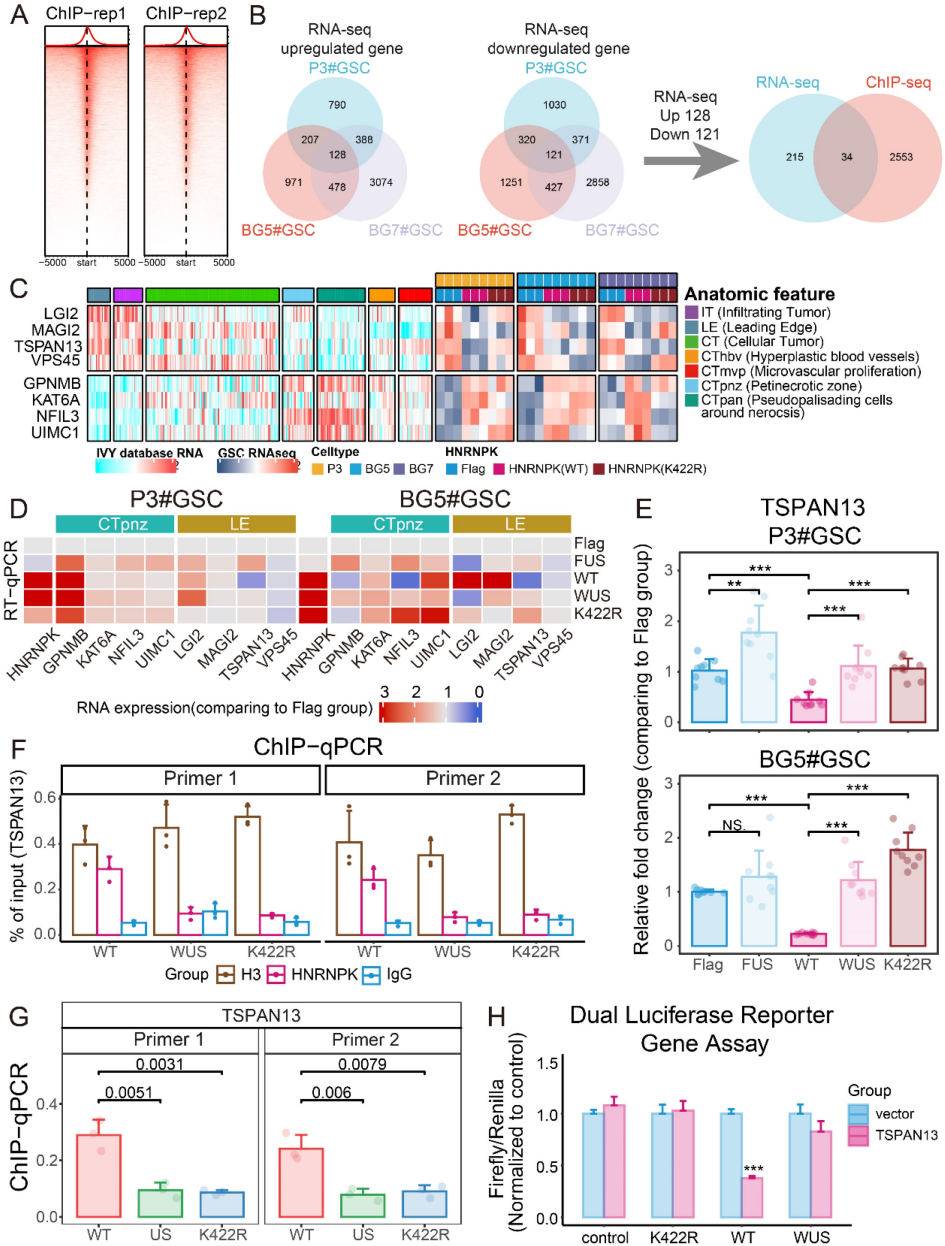
** HNRNPK inhibits the expression of TSPAN13.** A. Enrichment heatmap of the ChIP-seq results. B. Flowchart illustrating the process of screening for molecules regulated by HNRNPK transcription. C. Complex heatmap showing the RNA-seq expression of the molecules discovered by ChIP-seq and their localization in GBM tissues. D. RT-qPCR analysis of molecules in P3#GSCs and BG5#GSCs overexpressing Flag, FUS (Flag + UBE2I + SUMO1), WT (HNRNPK), WUS (HNRNPK (WT) + UBE2I + SUMO1), or K422R (HNRNPK). The data represent the average of replicates comparing the overexpression Flag group. E. RT-qPCR results of TSPAN13 in P3#GSCs or BG5#GSCs with overexpression of Flag, FUS (Flag + UBE2I +SUMO1), WT (HNRNPK), WUS (HNRNPK (WT) + UBE2I + SUMO1), or K422R (HNRNPK). Data are presented as the mean ± SD; n = 9. F. Barplot showing the ChIP-qPCR results of two primer sets targeting the promoter region of TSPAN13. H3 was used as a positive control, and IgG was used as a negative control. Data are presented as the mean ± SD. n = 3. G. Barplot displaying the results of ChIP-qPCR quantification. Data are presented as the mean ± SD; n = 3. H. Barplot of the dual luciferase reporter gene assay results. Data are presented as the mean ± SD; n = 3. *p < 0.05, **p < 0.01, ***p < 0.001, ****p ≤ 0.0001.

Because SUMO1 modification interferes with the binding of HNRNPK to DNA, we next conducted ChIP-seq to detect the sequences bound by HNRNPK (Figure 6A). We separately selected the molecules commonly up- and down-regulated in GSCs and intersected these with the findings from the ChIP-seq analysis (Figure 6B). The results showed that wild-type HNRNPK upregulated molecules located in the CTpnz region (GPNMB, KAT6A, NFIL3, and UMIC1) while inhibiting markers of infiltration and those on the leading edge, such as LGI2, MAGI2, TSPAN13, and VPS45 (Figure 6C). Further RT-qPCR experiments revealed that the expression of TSPAN13 was suppressed by HNRNPK, and that this process could be reversed by promoting intracellular SUMOylation modification by overexpressing SUMO1 and UBE2I (Figure 6D).

TSPAN13 is a marker of NPC-like cells 4. Data analysis suggests that TSPAN13 is predominantly highly expressed in the PN subtype and primarily localized at the leading edge and around the microvascular proliferation zone of GBM (Supplementary Figure 10A-D). Therefore, we speculate that HNRNPK can suppress the expression of TSPAN13 in cells, thereby inhibiting the transition of GSCs towards the PN subtype. Two primer sets were designed in the promoter region of TSPAN13, and ChIP-qPCR was used to assess the efficiency of HNRNPK binding to TSPAN13 (Figure 6F-G and Supplementary Figure 10E). The results showed that HNRNPK (WT) could bind to the promoter region of TSPAN13, while both mutations at the K422 site and the promotion of intracellular SUMOylation modification by overexpressing SUMO1 and UBE2I disrupted this process.

We further performed a luciferase reporter assay to clarify the impact of HNRNPK on TSPAN13 expression. The results demonstrated that HNRNPK (WT) significantly interfered with the expression of exogenous TSPAN13 and that this process could be disrupted by the overexpression of SUMO1 and UBE2I in cells (Figure 6H). We further knocked down TSPAN13 in P3 and BG5 cell lines and observed a significant increase in the phosphorylation levels of P38 and CREB within the cells after TSPAN13 knockdown (Supplementary Figure 10F).

### HNRNPK regulates cell proliferation and infiltration *in vivo*

By overexpressing WT or K422R mutant HNRNPK in GSCs, PDX models were constructed to mimic the *in vivo* situation. In all groups, 3 × 10^5^ GSCs were intracranially implanted. Significant tumor formation was observed 7 days after implantation with overexpressed HNRNPK (WT). The tumorigenic capacity was significantly reduced in the HNRNPK (K422R) treatment group (Supplementary Figure 11A-B). Additionally, the survival period of mice in the K422R group was noticeably improved (Supplementary Figure 11C-D). Our results are consistent with the current view that *in vivo* tumor formation is more challenging for PN-subtype GSCs 26.

**Figure 7:**
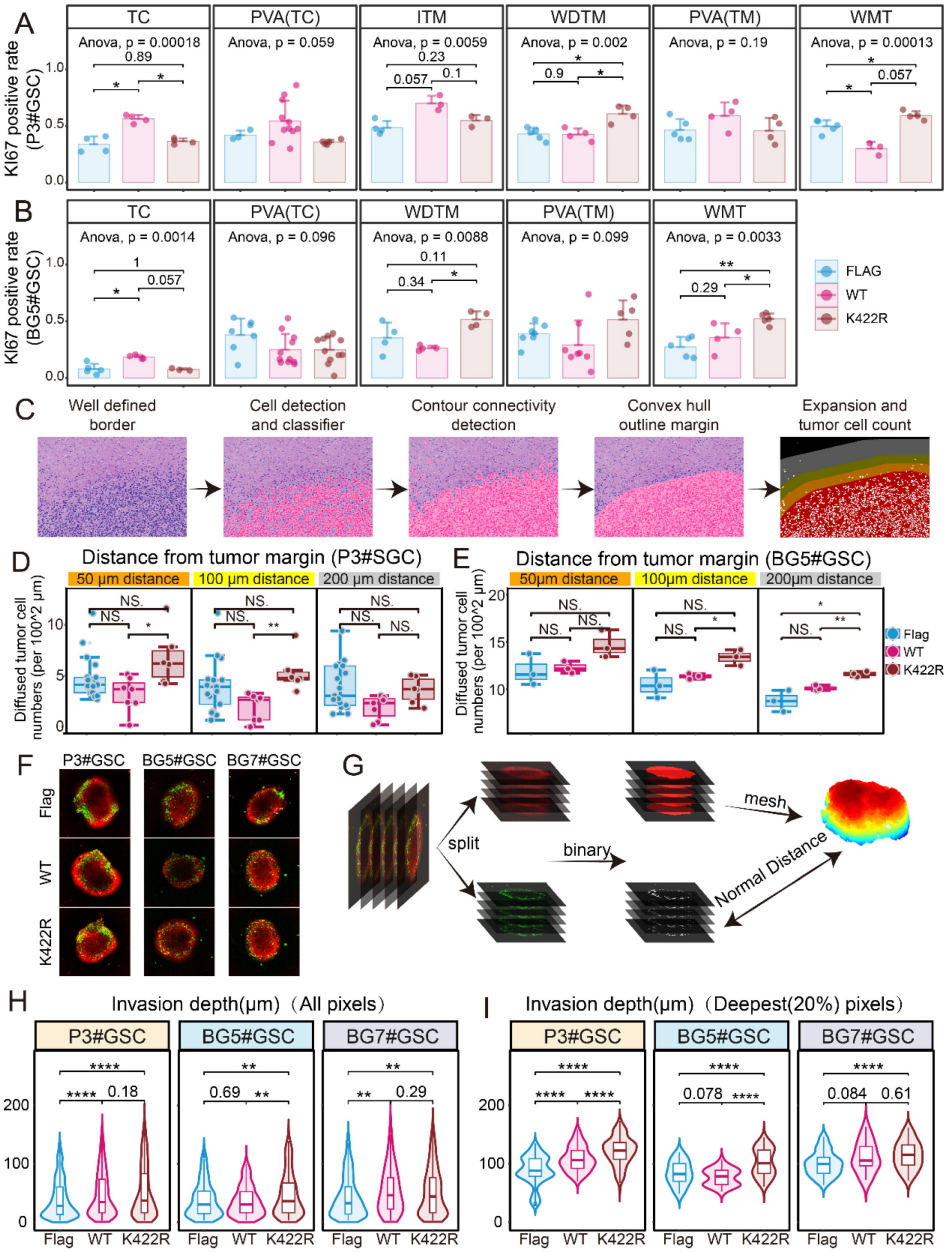
** HNRNPK regulated cell invasion and proliferation in different anatomic regions.** A. Ki67 positive rates in different anatomic regions of PDX models (xenograft intracranial P3#GSCs overexpressing HNRNPK (WT) or HNRNPK (K422R)). Data are presented as the mean ± SD. B. Ki67 positive rates in different anatomic regions of PDX models (xenograft intracranial BG5#GSCs overexpressing HNRNPK (WT) or HNRNPK (K422R)). Data are presented as the mean ± SD. C. Flowchart illustrating the data processing pipeline. D-E. Cell numbers different distances away from the tumor border; n ≥ 3. F. Distributions of GFP voxel (tumor cells) distances from the brain organoid surface among GSCs overexpressing Flag, HNRNPK (WT), or HNRNPK (K422R). G. Flowchart illustrating the data processing pipeline. H-I. Invasion depth of all GFP voxels (tumor cells) or the top 20% invasive GFP voxels (tumor cells) in GSCs (P3, BG5, and BG7) overexpressing Flag, HNRNPK (WT), or HNRNPK (K422R); n ≥ 5. *p < 0.05, **p < 0.01, ***p < 0.001, ****p ≤ 0.0001.

We observed that GSCs in different treatment groups exhibited varying proliferation abilities in different regions of the tissue (Supplementary Figure 11E-F). Tumors overexpressing HNRNPK (WT) showed a higher Ki67 positivity rate in the core regions, indicating increased proliferation in these areas. In contrast, tumors overexpressing the K422R mutant of HNRNPK exhibited a higher average Ki67 positivity rate at the well-defined tumor margin and white matter tract, suggesting enhanced proliferation in the tumor margin and infiltrating region (Figure 7A-B) 19. Hematoxylin and eosin (HE) staining of tissue sections were performed to analyze diffused tumor cells in the PDX model. The tumor boundary was delineated to differentiate infiltrative tumor cells at the tumor edge, and their distances from the tumor boundary were measured (Figure 7C). The number of infiltrative tumor cells in the different treatment groups was compared, revealing that the HNRNPK (K422R) treatment group exhibited a higher number of infiltrative tumor cells at the tumor edge (Figure 7D-E, Supplementary Figure 11G-H). Through three-dimensional reconstruction of multi-layer fluorescence scanning (Figure 7F-G) 28, we studied the invasive ability of GSCs in GBM-brain co-culture structures. The invasion into the interior of the organoid-like structures was found to be primarily by individual tumor cells, while the quantitative results also showed that GSCs overexpressing HNRNPK (K422R) exhibited stronger invasive abilities compared to GSCs overexpressing HNRNPK (WT) (Figure 7H-I).

## Discussion

Our investigation provides compelling evidence that cells exhibit a sophisticated response to environmental stressors by adjusting the levels of SUMO1 modification on HNRNPK. This adaptive mechanism is triggered by factors such as exposure to hypoxia, which are conditions commonly encountered in the tumor microenvironment. The modulation of SUMO1 levels on HNRNPK is a critical step in the cellular strategy to cope with these challenges, as it directly influences the protein's ability to bind to ssDNA. This binding is essential for the proper regulation of transcription, and any disruption in this process can have profound effects on cellular function. Specifically, our findings indicate that these changes in HNRNPK-ssDNA interactions play a crucial role in controlling the fate of GSCs, guiding their transition into distinct cellular states. This insight into the cellular response to environmental cues offers a deeper understanding of the complex biology of GBM.

Due to the inability to establish stable protein modifications *in vitro*, current research on protein modifications often relies on site mutations as substitutes. It is commonly accepted that mutations and modifications have opposite effects on protein function. However, the SPR assay proved that mutation and SUMO1 modification at the K422 site of HNRNPK have the same effects from the perspective of its DNA-binding ability. The changes in GSC subtypes driven by overexpression of HNRNPK (WT or K422R) were not restricted by GSC mutation features, which is in line with the results of multiple studies showing that different cellular states can undergo transition without being restricted by mutation status 5, 29-32. Sequencing of GSCs with overexpression of the HNRNPK (K422R) mutation confirmed the upregulation of infiltrating markers within the cells 32. The increased expression of infiltrating markers also aligns with the staining results of HNRNPK-SUMO1, which exhibits a higher HNRNPK-SUMO1 positivity rate in the infiltrating region of tumors. Therefore, we used the HNRNPK (K422R) mutation to investigate the effect of inhibiting the binding of HNRNPK to ssDNA after SUMO1 modification on cells.

As is currently known, MES-type cells are mostly located in the core of the tumor, whereas the infiltrating tumor cells are predominantly PN-type, indicating a close association between cell subtypes and anatomical location 33. It seemed that using anatomy features to describe the difference between these two groups is more accurate in our study, suggesting that the cellular state transition driven by SUMO1 modification of HNRNPK is associated with anatomical location. Currently, there is no consensus regarding the functional characteristics of different types of GSCs in terms of proliferation and invasion. Ichiro Nakano's study compared the proliferation abilities of MES and PN subtypes of GSCs *in vitro* and found that the MES subtype exhibited stronger proliferation, while several studies on the epithelial-mesenchymal transition suggested that the MES subtype has stronger invasive ability 34 35. In fact, our *in vitro* experimental studies also observed that cells of the MES/core subtype driven by wild-type HNRNPK exhibit stronger proliferation and invasive capabilities (results not shown in the article). However, our observations from animal experiments contradict the results obtained from *in vitro* experiments. Our study found that the invasion of GSCs into surrounding brain tissue predominantly occurs through single-cell infiltration and that GSCs transitioning to the PN/infiltration phenotype exhibit stronger invasion. Meanwhile, GSC proliferation is influenced by cell type and anatomical location. GSCs transitioning to the MES/core subtype exhibit increased proliferation in the core region of the tumor, while GSCs transitioning to the PN/infiltration subtype show enhanced proliferation around white matter tracts and well-defined margins. This discrepancy in the observed proliferation ability suggests a correlation between cell type and tissue localization. As we know, Verhaak's single-cell glioblastoma analysis suggested that NPC/OPC-like cells have higher proliferative capacity 4. NPC/OPC-like cells are the main constituent cells of PN-type GBM. Moreover, Frank Winkler's study revealed that NPC-like cells primarily contribute to invasion in GBM *in vivo* 14. Current studies are exploring the therapeutic potential of targeting heterogeneity in GBM treatment, and the PN subtype is believed to have a better prognosis and lower drug resistance. Some studies have considered promoting tumor transformation towards the PN subtype to improve patient outcomes. Jin's study proved simultaneous treatment of both subtypes are more effective than any treatment targeting a single subtype and proposed simultaneous treatment for PN and MES subtypes 36. Although our research also found that GSC-bearing mice undergoing transformation towards the PN/infiltration state had significantly better prognoses, this was accompanied by an increase in tumor cells infiltrating normal brain tissue, which undoubtedly increased the difficulty of achieving complete surgical resection. Treatment of PN-type GBM may require more extensive resection. As the transformation of GSCs towards the MES direction enhances their tumorigenicity, transitioning towards the PN type, although weaker in tumorigenicity, enhances their invasiveness *in vivo*. Therefore, promoting their transformation into the PN subtype, then suppressing invasiveness by regulating TSPAN13, might be a potential treatment approach. Exploring TSPAN13's role in maintaining the neuronal or neural precursor cell state, as well as its regulation of glutamatergic or CREB signaling pathways, may provide deeper insights into its function in tumor invasion 14 37. However, the research did not delve deeply into the exploration. Further study is needed in future research.

Although it is recognized that GBM is a heterogeneous tumor, the current understanding of how the tumor microenvironment determines cellular states is limited. In our study, we proved that external factors such as hypoxia can decrease the level of SUMO1 modification of HNRNPK, which may partially explain why cells in different locations have different states. Anne Dirkse's study also found that hypoxia could induce phenotypic adaptation, and heterogeneity instructed by the microenvironment provides a growth advantage *in vivo* 31. However, the functions of SUMOylation extend beyond this. Protein SUMOylation not only affects DNA binding but can also recruit interacting proteins to exert additional functions. SUMO proteins can engage in non-covalent interactions with SUMO-interacting motifs (SIMs) in other proteins 38. Through co-IP experiments, we found that the mutated HNRNPK had a reduced number of bound proteins, although further investigation is needed to determine the specific reasons for this. Moreover, it remains to be determined whether the decrease is due to reduced DNA binding capacity leading to decreased protein binding or whether mutation directly interferes with HNRNPK's interaction with other proteins. Given the complexity of the *in vivo* environment, it remains to be investigated which factors can regulate the level of cellular SUMO1 modification. The influence of neighboring cells, such as immune cell infiltration or neuronal stimulation, exogenous stimuli, such as radiation and chemotherapy, or the acidity or alkalinity of the cellular microenvironment may affect the level of modification and regulate cellular state transitions. Further research is necessary to explore these factors in depth.

## Supplementary Material

Supplementary figures, tables, and information.

## Abbreviations

GBM: Glioblastoma multiforme
GSC: glioblastoma stem cell
IDH: isocitrate dehydrogenase
PN: proneural
MES: mesenchymal
CL: classical
AC-like: astrocyte-like
MES-like: mesenchymal-like
NPC-like: neural progenitor-like
OPC-like: oligodendrocyte progenitor-like
PFS: progression-free survival
PDX: Patient-derived tumor xenograft
WMT: white matter tract
SPR: surface plasmon resonance
WT: wildtype
2D-enrichment: two-dimensional enrichment
UV: ultraviolet
HE: Hematoxylin and eosin
GSVA: Gene Set Variation Analysis
GSEA: Gene Set Enrichment Analysis
ChIP: Chromatin immunoprecipitation
