# Supplementary material for: SUMOylation modification of HNRNPK at the K422 site promotes invasion in glioblastoma
Source: Int J Biol Sci. 2024 Oct 21;20(14):5715–30. doi: 10.7150/ijbs.102051 (PMC11528450; doi:10.7150/ijbs.102051)
Supplement: Supplementary file 1 — Supplementary figures, tables, and information. [file ijbsv20p5715s1.pdf]

SUMOylation modification of HNRNPK at the K422 site promotes invasion in glioblastoma

Wenguo Zhao[1,2], Jiazheng Wang[1,2], Feihu Zhao[1,2], Yaquan Li[1,2], Zhuo Li[1,2], Xingang Li[1,2]*, Anjing Chen[1,2]*

[1] Department of Neurosurgery, Qilu Hospital, Cheeloo College of Medicine and Institute of Brain and Brain-Inspired Science, Shandong University, Jinan, 250012, China

[2] Jinan Microecological Biomedicine Shandong Laboratory and Shandong Key Laboratory of Brain Health and Function Remodeling, Jinan, 250017, China

*These authors contributed equally to this work as senior authors.

This Supplementary file includes:

Materials and Methods

Supplementary Figures. 1 to 11

Tables

Supplementary Text

Materials and Methods

Transfection of cells

GSCs were transfected with lentiviruses to overexpress HNRNPK (WT) or HNRNPK (K422R) tagged with Flag and GFP. Empty vectors with Flag and GFP tags were used as control groups. After lentiviral transduction, puromycin was used for drug selection. Fluorescence was observed to determine the transfection efficiency. Plasmids were used for the exogenous overexpression of SUMO1, UBE2I. All sequences are presented in Supplementary Text.

Western blotting

Cells were centrifuged and washed with PBS, followed by lysis on ice for 15 min using RIPA lysis buffer (R0010, Solarbio) with 10% NEM. Protein expression was quantified using the BCA method. SDS-PAGE was performed, and proteins were transferred onto a PVDF membrane. The membrane was then blocked with milk at room temperature for 1 h, followed by overnight incubation with the primary antibody on a rocking platform at 4°C. After washing three times with TBST, the membrane was incubated with the secondary antibody at room temperature for 1 h. Immobilon Western HRP (WBKLS0500, Millipore) was used for detection. The list of antibodies can be found in Supplementary table 1. Quantitative analysis of the Western blot images was performed using ImageJ software.

Immunohistochemistry data analysis

All immunofluorescence slides were imported into QuPath for processing. An artificial neural network was employed for tumor and normal cell classification. All slides underwent color normalization and annotation of tumor and normal regions to eliminate batch effects. The tumor core region was defined as an area > 500 μm from any edge, and the perivascular area extended 30 μm outward from the vascular edge. All white matter tracts (WMTs) were annotated in the corpus callosum region. The invasive margin refers to the protruding part of the tumor’s main edge. All annotated regions used for statistical analysis were independently reviewed by two experienced pathologists. For image processing and analysis, the annotated region images were imported into Spyder and processed using OpenCV. Contour connectivity was used for edge analysis to detect the connectivity of tumor cells. The convex hull was applied to fit the edges, and extensions of 50, 100, and 200 μm were used to count diffused cell numbers at the edges.

GBM-brain organoid co-culture invasion assay analysis

All data were analysis with openCV and skimage. The images underwent brightness adjustment and were then subjected to binary processing. Connected component analysis techniques were applied to extract connected regions from the red channel of the image. The pixel values corresponding to these regions are preserved in the resulting color image. Subsequently, a surface model is generated using triangulation. The distances from green pixels to the reconstructed red surface are calculated using normal distance computation. The Open3D library is utilized for reconstructing the 3D model in this process.

Chromatin immunoprecipitation (ChIP)

The ChIP experiment followed the instructions provided in the SimpleChIP® Enzymatic Chromatin IP Kit (9003, CST). Cells were fixed with formaldehyde and lysed, and chromatin was digested using micrococcal nuclease to obtain chromatin fragments associated with nucleosomes. ChIP was performed using anti-FLAG M2 magnetic beads (M8823, Sigma) and ChIP-grade protein G magnetic beads. After reversing the protein-DNA cross-links, DNA was purified using DNA purification spin columns. ChIP-seq analysis was performed as described in the sequencing data data analysis section.

Sequencing data processing

The GRCh38.primary_assembly.genome.fa.gz and gencode.v41.annotation.gtf.gz files were used as the reference genome and annotation. RNA-seq data was quality controlled using fastqc and then aligned and quantified using STAR. The resulting files were converted to bw format using deeptools for visualization. GATK4 was employed for mutation detection in RNA-seq data. ChIP-seq data was aligned using bowtie2, and peak calling was performed using MACS2 with no Input parameter. The results were annotated using CHIPseeker. Visualization was done using enrichedheatmap package.

Weighted Gene Co-expression Network Analysis (WGCNA)

The top 10,000 rows with the highest Median Absolute Deviation (MAD) were selected for further analysis. The scale-free network fit was calculated for different soft-thresholds using the pickSoftThreshold function. The soft-threshold value was chosen based on the analysis of the resulting scale-free fit index plot and the mean connectivity plot. Modules were detected using the blockwiseModules function. The soft-threshold value determined from the previous step was used in this process. Specific modules of interest were selected for subsequent gene function annotation.

Data analysis

The single-cell data was obtained from GSE159416. IVY data was acquired from http://glioblastoma.alleninstitute.org/. The complexheatmap package was used to generate heatmaps. Aneuploidy score, MSI score, RAGNUM hypoxia score, mRNAsi score, Stromal score, Immune score, ESTIMATE score, tumor purity and ABSOLUTE score were calculated according to previously published methods. The single-cell data was processed using the Seurat package, selecting GBM samples with cell numbers greater than 6000, filtering out mitochondrial genes, conducting CCA to identify highly variable genes, and annotating using singleR. InferCNV was used for tumor cell annotation with immune cells as a reference, and harmony was applied to integrate the data. After filtering for tumor cells, AddModuleScore was used to score based on molecular markers, with the highest score assigned to the corresponding cluster. t-SNE was used for dimensionality reduction and visualization. GO, REACTOME, HALLMARK, and other datasets were downloaded from the MSigDB database, and 2D-enrichment analysis was performed using the Perseus software.

Supplementary Figures

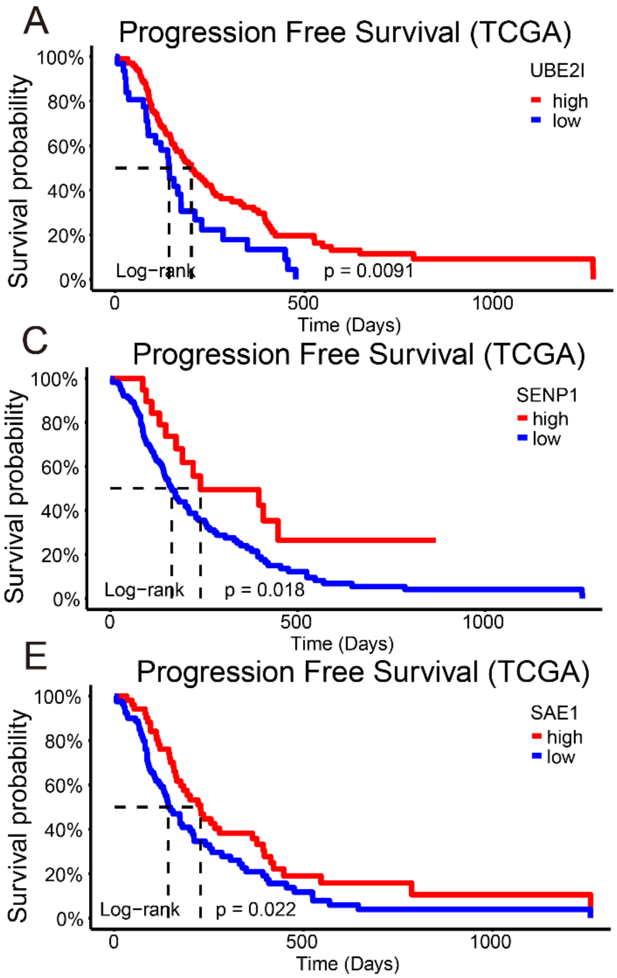

Supplementary Figure 1. Survival analysis of SUMO1-related molecules. A-F. Survival (progression free survival) analysis of SUMO-related molecules in GBM.

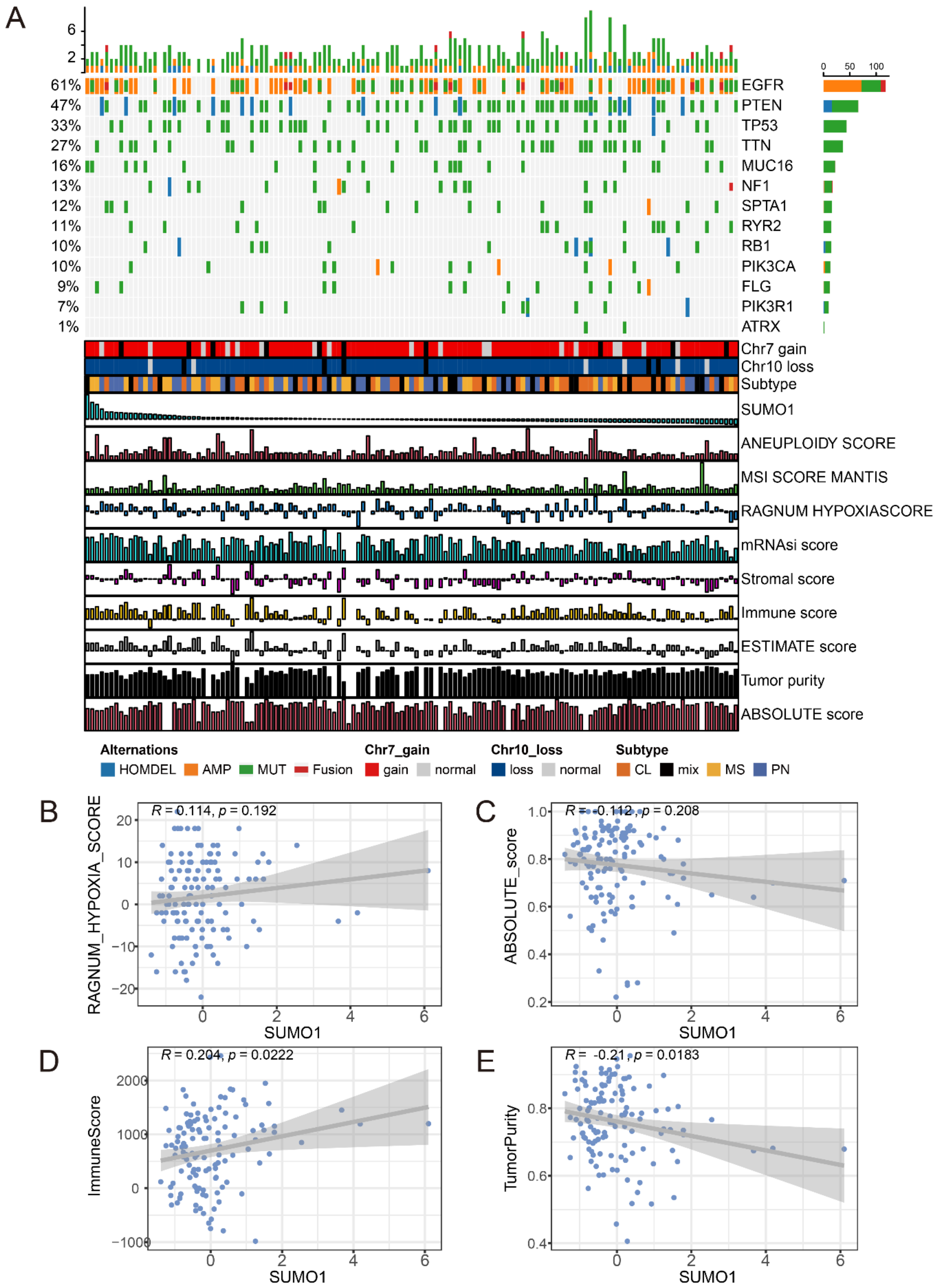
A
61% EGFR
47% PTEN
33% TP53
27% TTN
16% MUC16
13% NF1
12% SPTA1
11% RYR2
10% RB1
10% PIK3CA
9% FLG
7% PIK3R1
1% ATRX
Chr7 gain
Chr10 loss
Subtype
SUMO1
ANEUPLOIDY SCORE
MSI SCORE MANTIS
RAGNUM HYPOXIASCORE
mRNAsi score
Stromal score
Immune score
ESTIMATE score
Tumor purity
ABSOLUTE score
Alternations HOMDEL AMP MUT Fusion
Chr7_gain gain normal
Chr10_loss loss normal
Subtype CL mix MS PN
B
R = 0.114, p = 0.192
RAGNUM_HYPOXIA_SCORE
SUMO1
C
R = -0.112, p = 0.208
ABSOLUTE_score
SUMO1
D
R = 0.204, p = 0.0222
ImmuneScore
SUMO1
E
R = -0.21, p = 0.0183
TumorPurity
SUMO1

Supplementary Figure 2. Data analysis of SUMO1. A. Complexheatmap of GBM sample mutation status, chromosome structure, subtype, expression of SUMO1, and GSVA scores. Data were obtained from TCGA. B-E. Correlation analysis between SUMO1 and corresponding GBM scores.

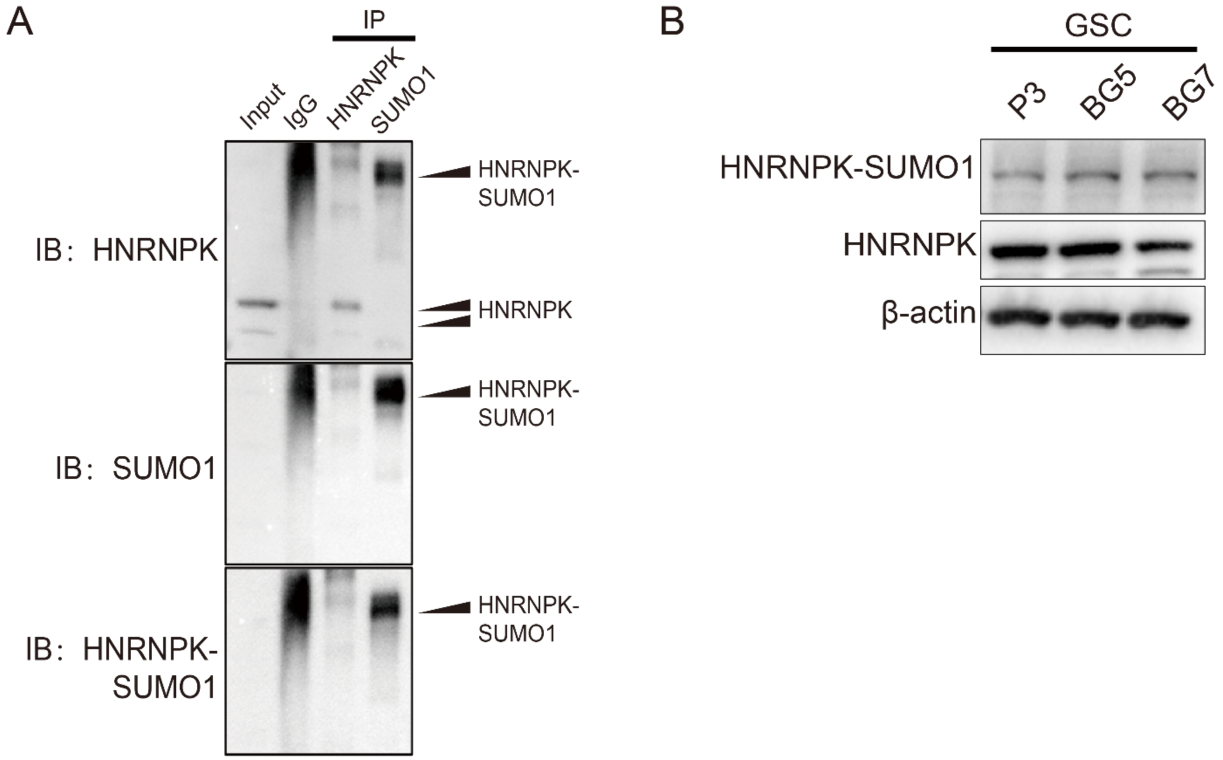

Supplementary Figure 3. HNRNPK-SUMO1 expression validation. A. Western blotting of the co-immunoprecipitation results of HNRNPK or SUMO1. B. Expression of HNRNPK and HNRNPK-SUMO1 proteins in three types of GSCs (P3, BG5, and BG7).

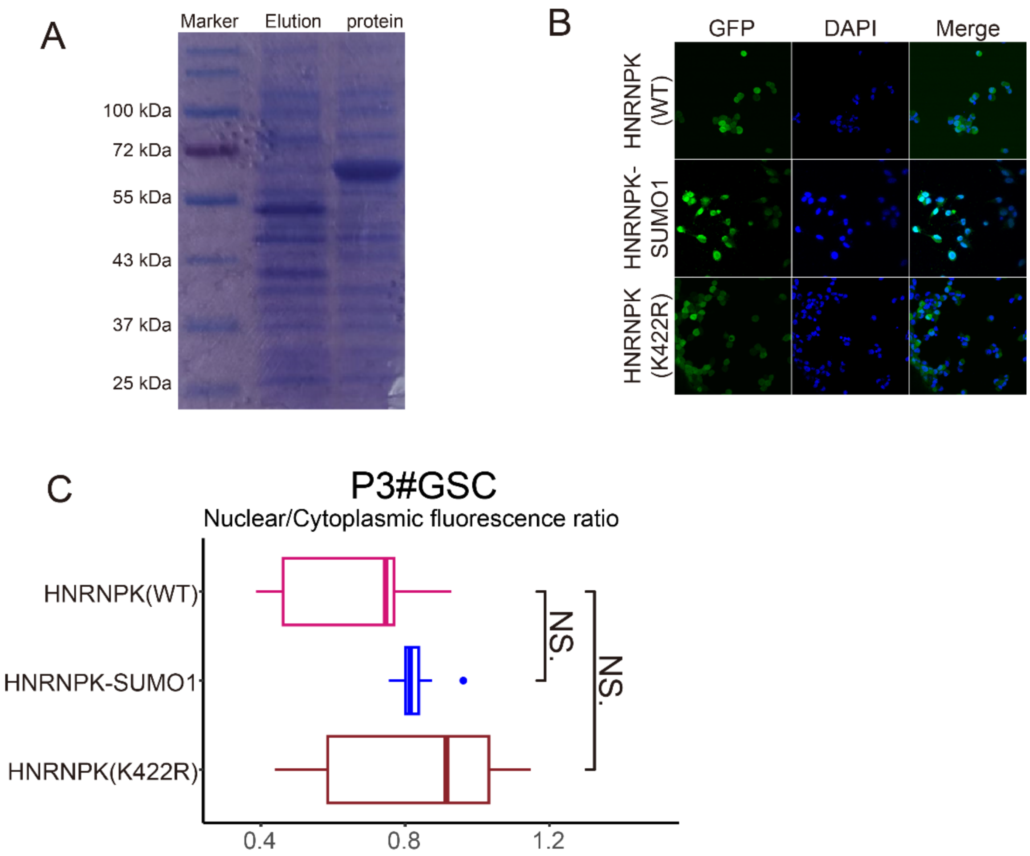

Supplementary Figure 4. Localization of HNRNPK-SUMO1 in cells. A. Coomassie bright blue staining of the purified HNRNPK (WT). The solution remaining after purification was collected as the elution fraction. B. Distribution of HNRNPK (WT), HNRNPK-SUMO1, or HNRNPK (K422R) in the nuclear or cytoplasmic fraction of P3#GSCs. C. Distribution of HNRNPK (WT) or HNRNPK (K422R) in the nuclear or cytoplasmic fraction of P3#GSCs.

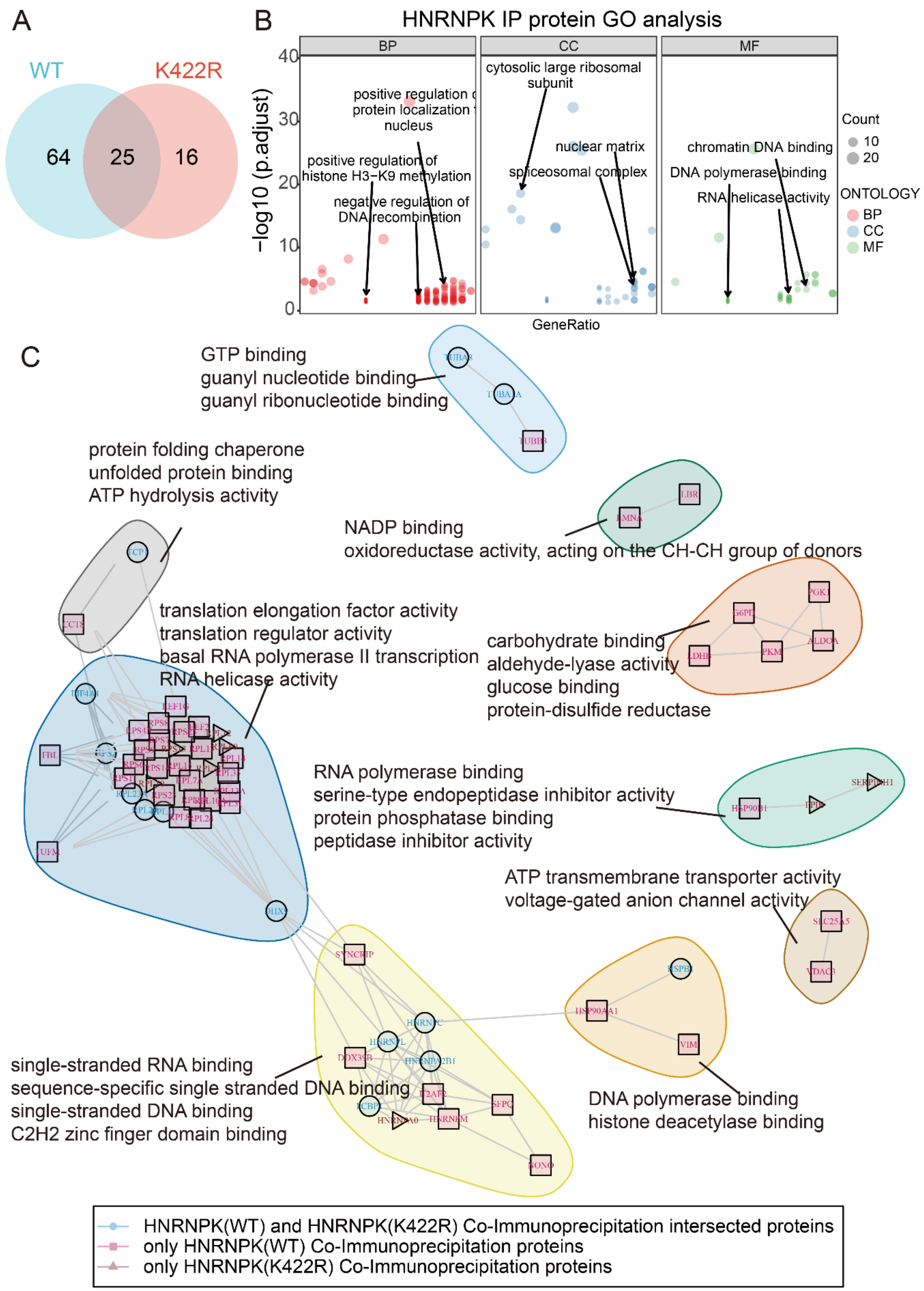
A
WT
K422R
64
25
16
B
HNRNPK IP protein GO analysis
BP
CC
MF
−log10 (p.adjust)
40
30
20
10
0
GeneRatio
positive regulation of protein localization to nucleus
positive regulation of histone H3−K9 methylation
negative regulation of DNA recombination
cytosolic large ribosomal subunit
nuclear matrix
spliceosomal complex
chromatin DNA binding
DNA polymerase binding
RNA helicase activity
Count
10
20
ONTOLOGY
BP
CC
MF
C
GTP binding
guanyl nucleotide binding
guanyl ribonucleotide binding
protein folding chaperone
unfolded protein binding
ATP hydrolysis activity
NADP binding
oxidoreductase activity, acting on the CH-CH group of donors
translation elongation factor activity
translation regulator activity
basal RNA polymerase II transcription
RNA helicase activity
carbohydrate binding
aldehyde-lyase activity
glucose binding
protein-disulfide reductase
RNA polymerase binding
serine-type endopeptidase inhibitor activity
protein phosphatase binding
peptidase inhibitor activity
ATP transmembrane transporter activity
voltage-gated anion channel activity
single-stranded RNA binding
sequence-specific single stranded DNA binding
single-stranded DNA binding
C2H2 zinc finger domain binding
DNA polymerase binding
histone deacetylase binding
HNRNPK(WT) and HNRNPK(K422R) Co-Immunoprecipitation intersected proteins
only HNRNPK(WT) Co-Immunoprecipitation proteins
only HNRNPK(K422R) Co-Immunoprecipitation proteins

Supplementary Figure 5. Co-immunoprecipitation results of HNRNPK. A. Venn diagram of protein co-immunoprecipitation with HNRNPK (WT) or HNRNPK (K422R). B. GO analysis of protein co-immunoprecipitated with HNRNPK (WT). C. Enrichment analysis and cluster of proteins that were co-immunoprecipitated by HNRNPK (WT) or HNRNPK (K422R).

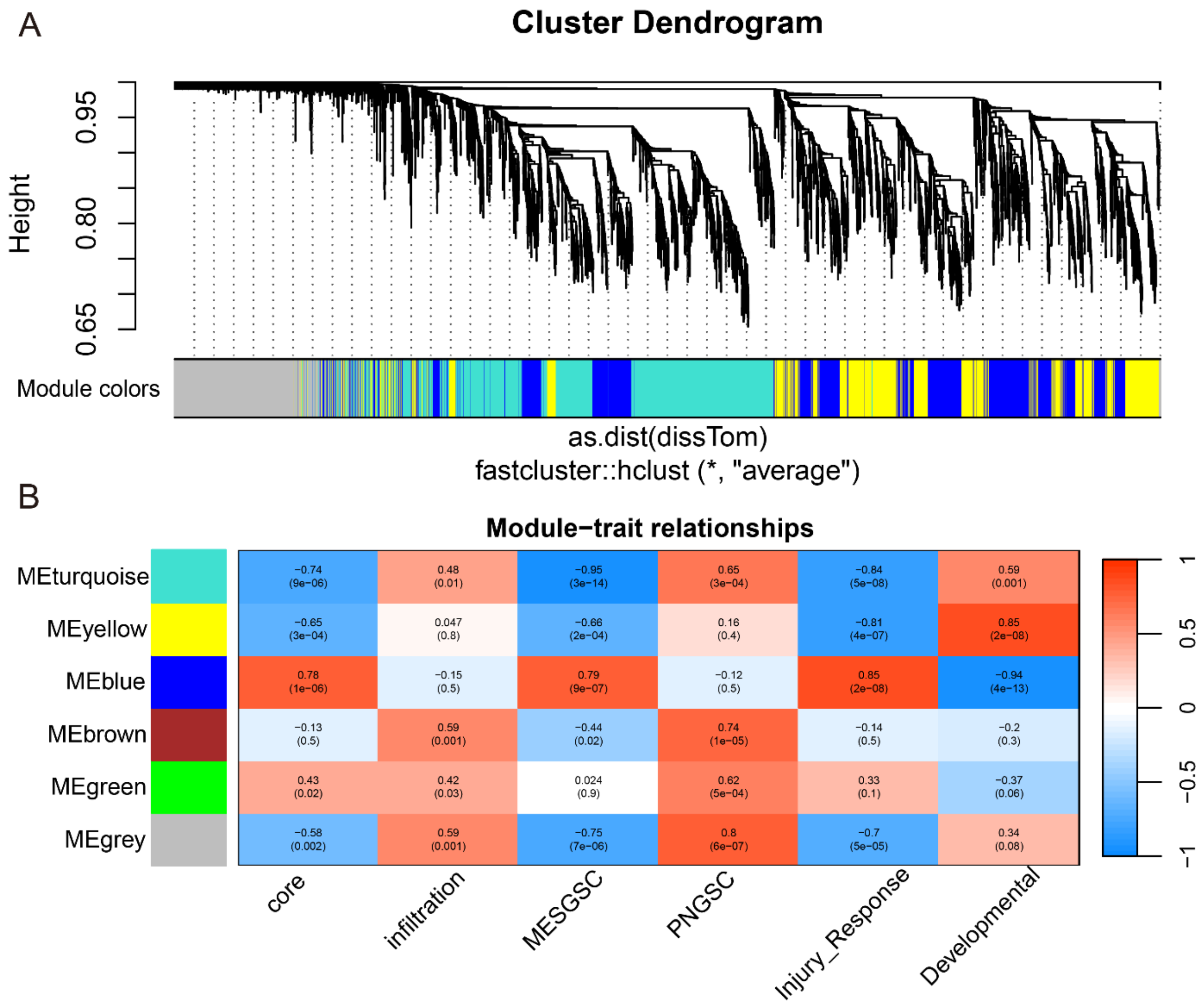

Supplementary Figure 6. WGCNA analysis of RNA-seq data. A. Cluster dendrogram of RNA-seq data. B. Correlation analysis of module and trait.

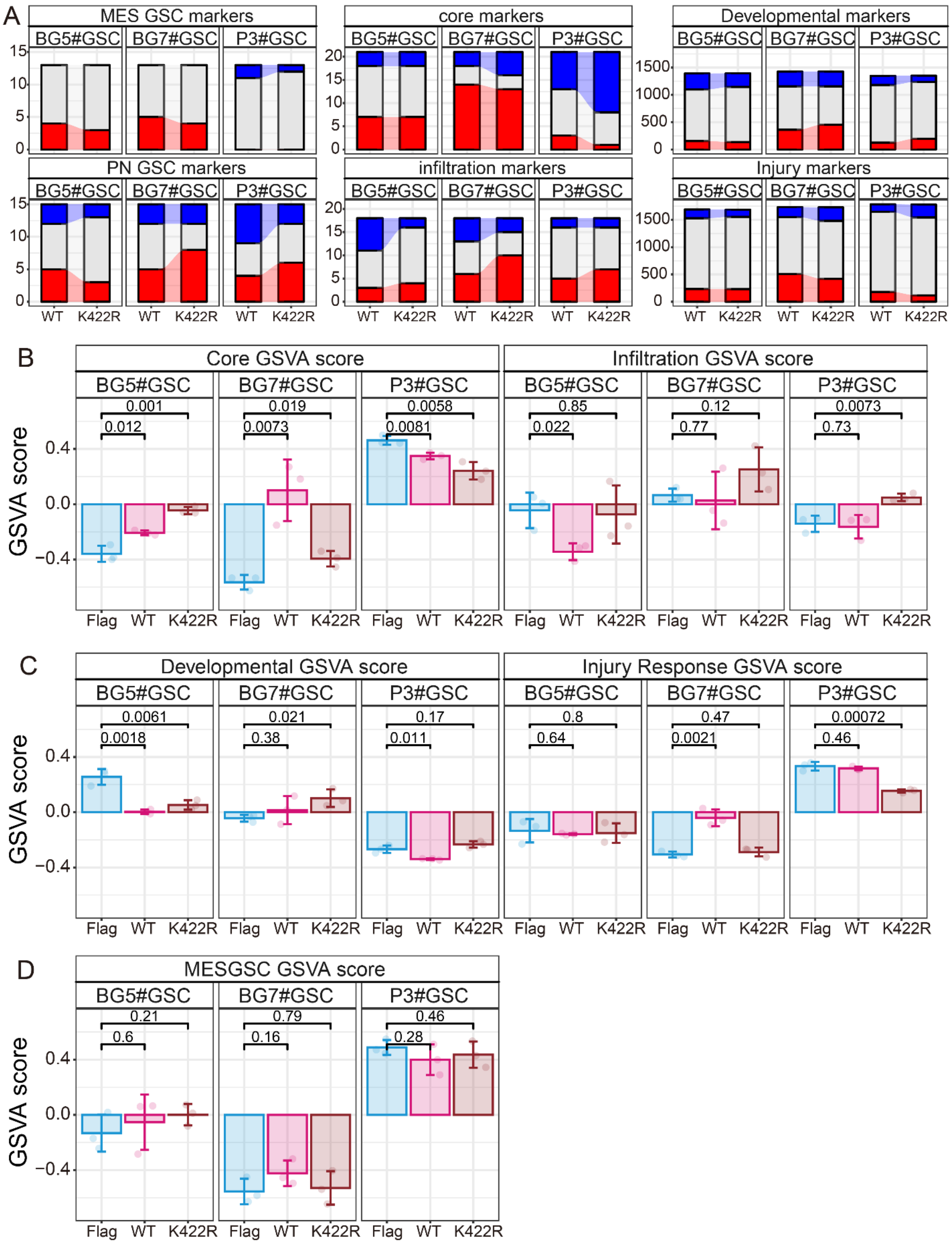
A
MES GSC markers
core markers
Developmental markers
PN GSC markers
infiltration markers
Injury markers
BG5#GSC
BG7#GSC
P3#GSC
WT
K422R
B
Core GSVA score
Infiltration GSVA score
GSVA score
Flag
WT
K422R
C
Developmental GSVA score
Injury Response GSVA score
D
MESGSC GSVA score

Supplementary Figure 7. Differentially expressed molecules and function of GSCs between wild-type and mutant HNRNPK. A. Alluvial plot illustrates the number of upregulated or downregulated genes in GSC overexpressing HNRNPK (WT) or HNRNPK (K422R). B-D. Boxplot presents the RNAseq GSVA scoring results of GSCs (P3, BG5, and BG7) overexpressing Flag, WT (HNRNPK), or K422R (HNRNPK).

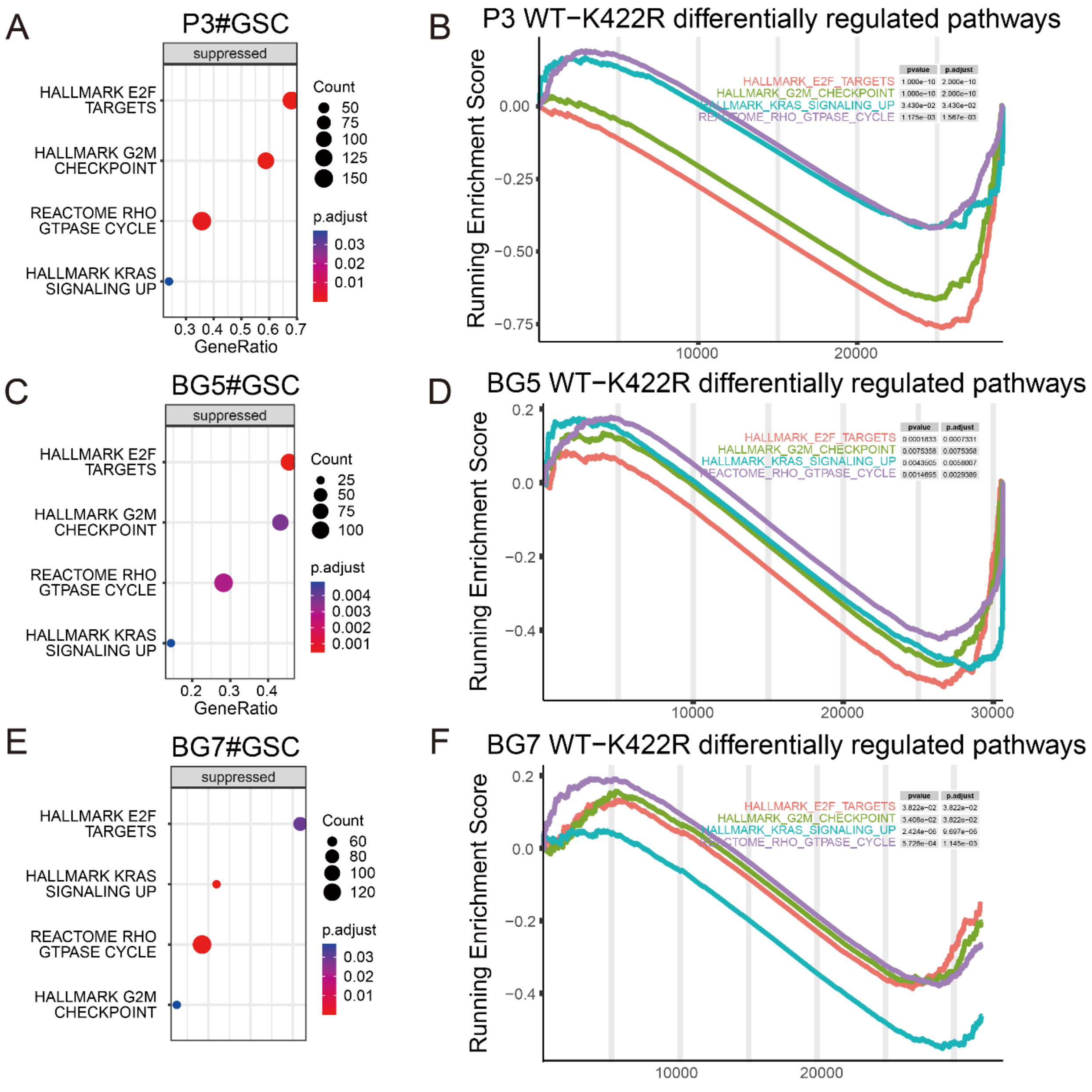

Supplementary Figure 8. Differential functions of GSCs regulated by HNRNPK. A-F. GSEA demonstrating the differential regulatory pathways with GSCs overexpressing HNRNPK (WT) or HNRNPK (K422R).

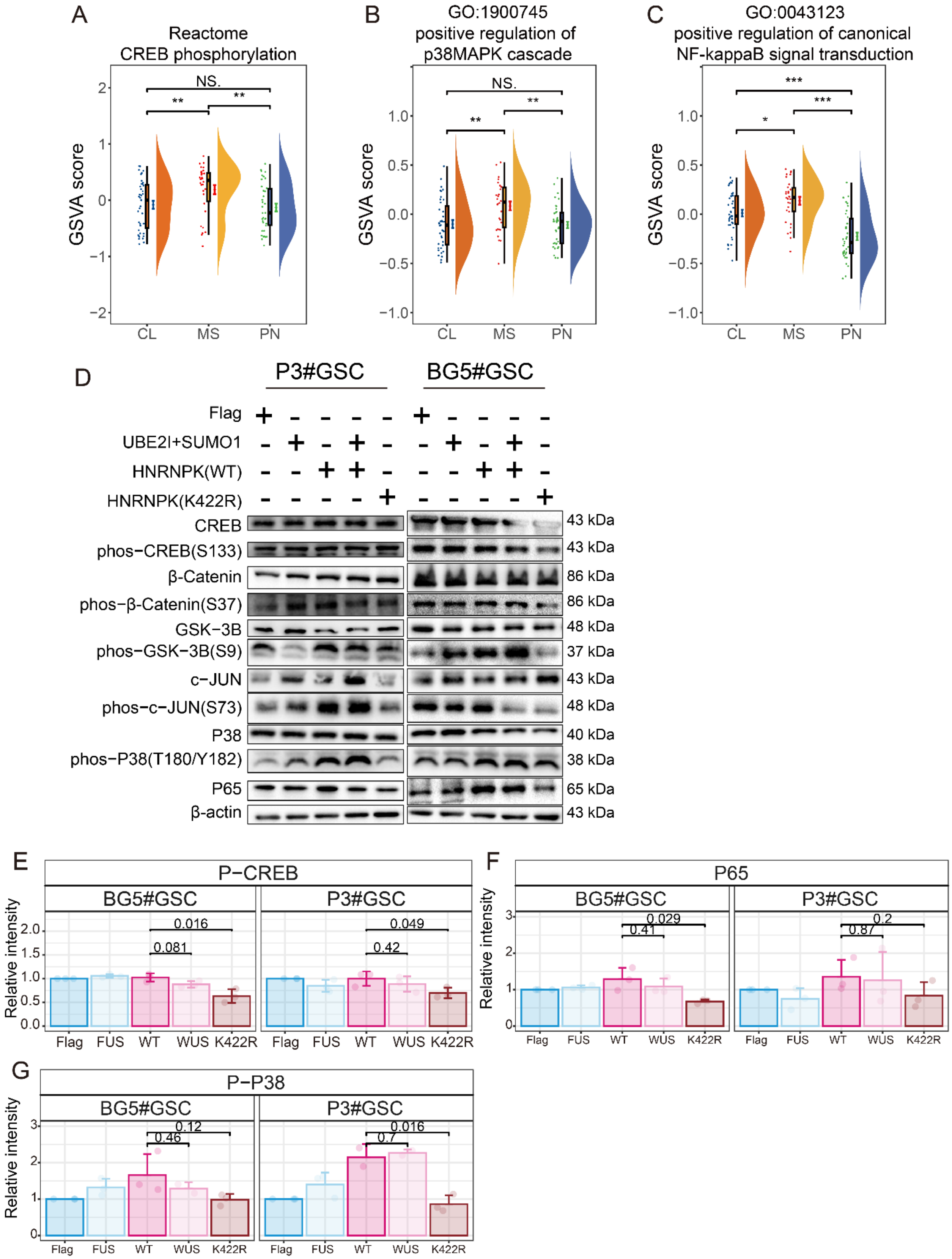
A
Reactome CREB phosphorylation
B
GO:1900745 positive regulation of p38MAPK cascade
C
GO:0043123 positive regulation of canonical NF-kappaB signal transduction
GSVA score
CL
MS
PN
NS.
D
P3#GSC
BG5#GSC
Flag
UBE2I+SUMO1
HNRNPK(WT)
HNRNPK(K422R)
CREB
phos−CREB(S133)
β-Catenin
phos−β-Catenin(S37)
GSK−3B
phos−GSK−3B(S9)
c−JUN
phos−c−JUN(S73)
P38
phos−P38(T180/Y182)
P65
β-actin
43 kDa
86 kDa
48 kDa
37 kDa
40 kDa
38 kDa
65 kDa
E
P−CREB
F
P65
G
P−P38
Relative intensity
Flag
FUS
WT
WUS
K422R

Supplementary Figure 9. Functional analysis of signaling pathways activated by HNRNPK. A. GSVA score of the CREB phosphorylation in different subtypes (CL, MES, PN) of GBM. B. GSVA score of the p38MAPK cascade in different subtypes (CL, MES, PN) of GBM. C. GSVA score of canonical NF-kappaB signal transduction in different subtypes (CL, MES, PN) of GBM. D. Western blotting image of P3#GSCs and BG5#GSCs detecting signaling pathway-associated proteins and phosphorylated proteins. E-G. Barplot of the protein's western blotting quantification. Data are presented as the mean ± SD; n = 3. **p* < 0.05, ***p* < 0.01, ****p* < 0.001, *****p* ≤ 0.0001.

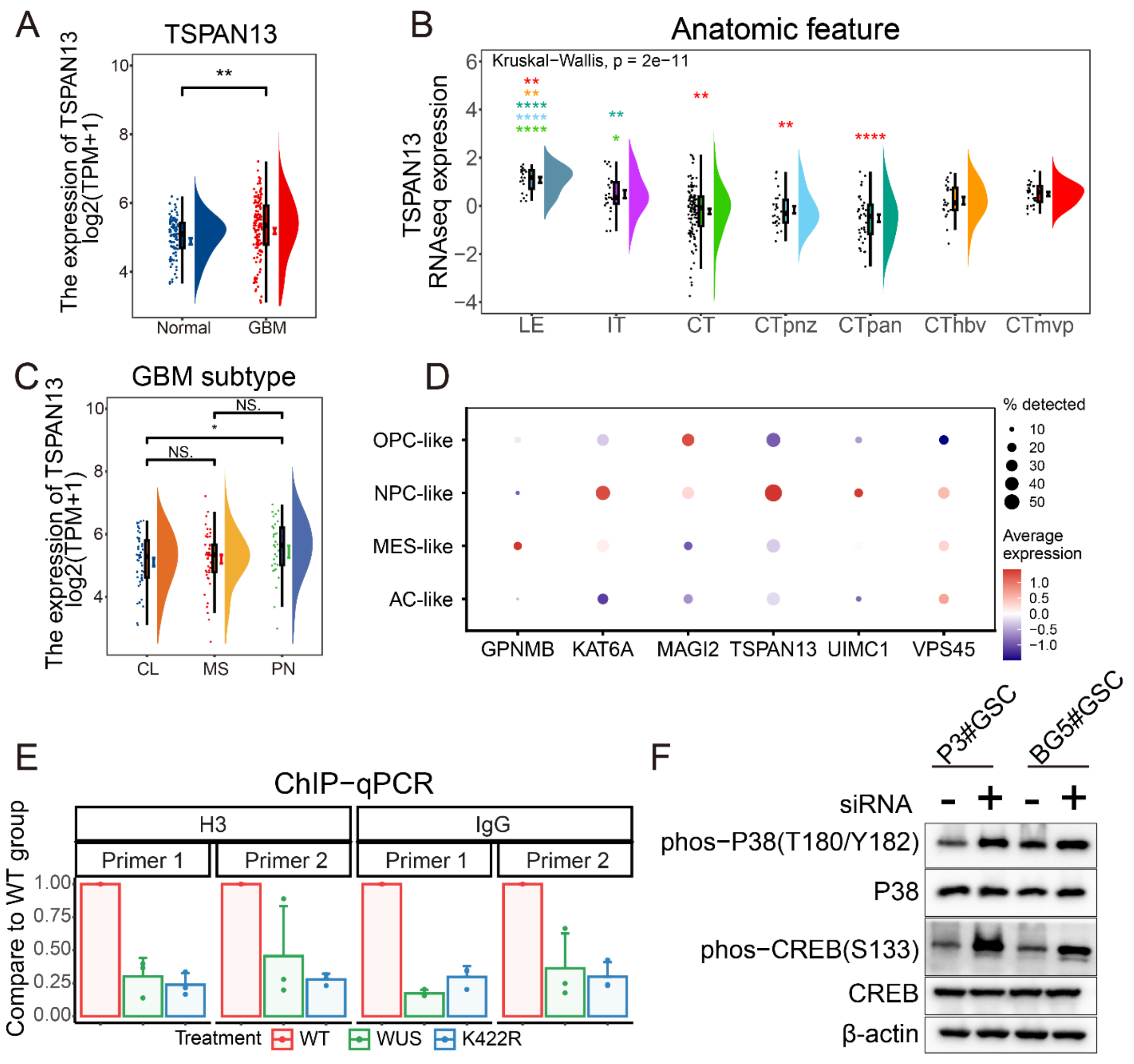

Supplementary Figure 10. Data analysis of TSPAN13. A. Expression of TSPAN13 in GBM and normal brains. B. Expression of TSPAN13 in different anatomic tumor regions. The data were obtained from IVY. C. TSPAN13 expression in different subtypes (CL, MES, PN) of GBM. D. Dotplot showing

the expression of MAGI2, TSPAN13, VPS45, GPNMB, KAT6A, and UIMC1 in different single cell types. E. ChIP-qPCR results of two primer sets targeting the promoter region of TSPAN13. Cells overexpressing HNRNPK (WT) were used as the control group. Data are presented as the mean ± SD; n = 3. F. Western blotting of proteins in P3#GSCs and BG5#GSCs after TSPAN13 knockdown. *$p < 0.05$, **$p < 0.01$, ***$p < 0.001$, ****$p \leq 0.0001$.

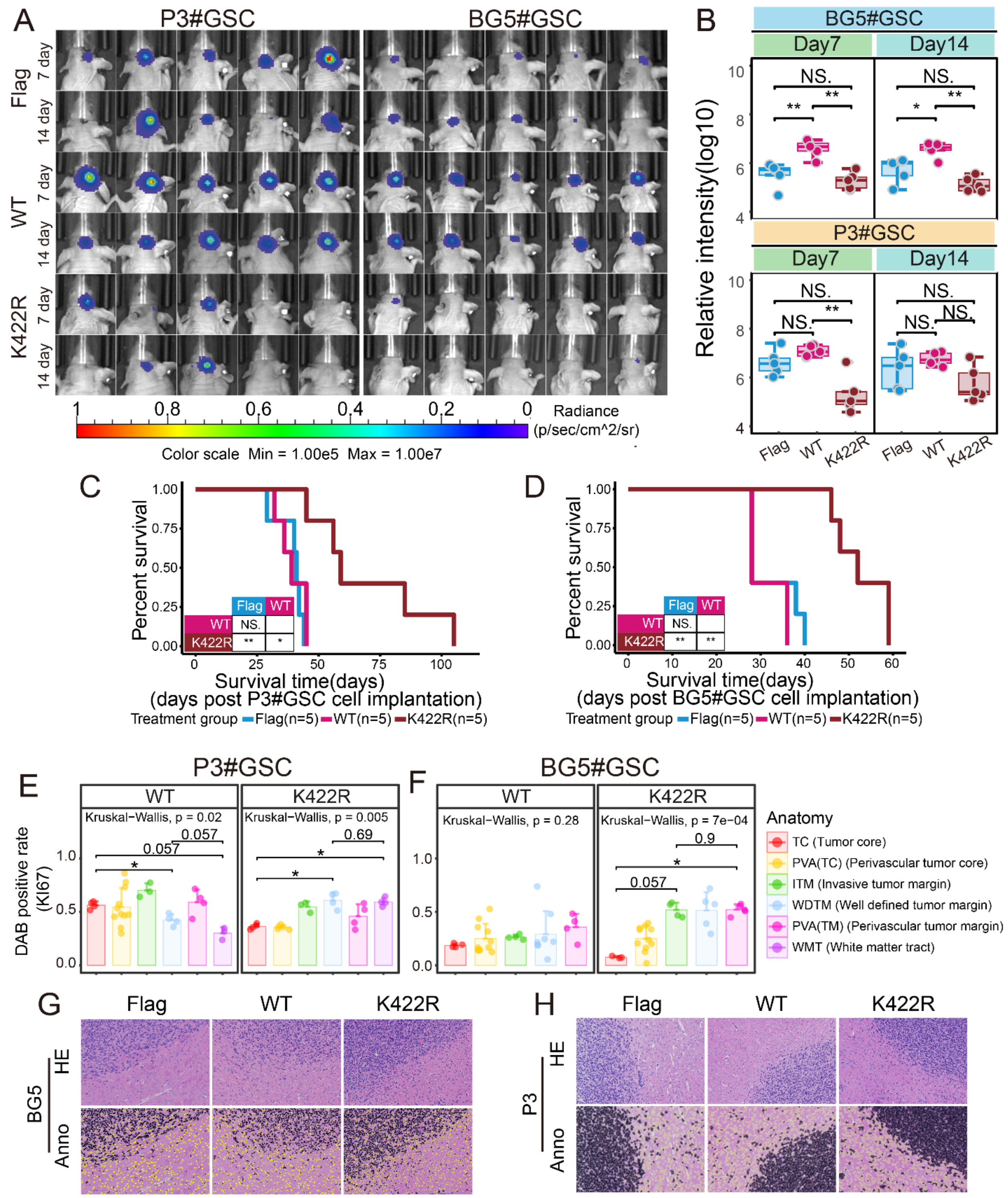

Supplementary Figure 11. Analysis of mouse PDX model. A. Bioluminescence imaging of xenograft intracranial P3#GSCs or BG5#GSCs overexpressing Flag, HNRNPK (WT), or HNRNPK (K422R); n = 5. B. Relative intensity of bioluminescence images at days 7 and 14. C. Survival curve

of the P3#GSC PDX model. D. Survival curve of the BG5#GSC PDX model. E-F. Ki67 positive rates in PDX models subjected to different treatments (xenograft intracranial P3#GSCs or BG5#GSCs overexpressing Flag, HNRNPK (WT), or HNRNPK (K422R)). Data are presented as the mean ± SD. G-H. HE and HNRNPK-SUMO1 DAB staining of tissue sections (xenograft intracranial P3#GSCs overexpressing HNRNPK (WT) or HNRNPK (K422R)). *p < 0.05, **p < 0.01, ***p < 0.001, ****p ≤ 0.0001.

Tables

Table S1

SUMOylated protein list						
ABAT	CALR	EZR	H4-16	MAOB	PLP1	SYNCRIP
ACAT1	CALU	FASN	HBA1	MAP1B	PPIA	SYNJ1
ACO2	CAMK2A	FGB	HBB	MAP2	PPIB	SYNPO2
ACRBP	CANX	FGG	HBD	MAP4	PPM1B	SYPL1
ACRV1	CAPN1	FHL1	HK1	MAPT	PRDX1	TECPR2
ACSL3	CCT2	FKBP4	HNRNPA1	MARCKS	PRDX2	TF
ACTN1	CCT4	FLNA	HNRNPA2B1	MBP	PRDX3	TGM2
ADH5	CCT6A	FN1	HNRNPC	MDH1	PRDX6	TKT
AKR1B1	CCT7	FUS	HNRNPK	MDH2	PRKCA	TLN1
ALB	CCT8	GAPDH	HNRNPL	MIF	PRKCSH	TNPO2
ALDH6A1	CFL1	GDI1	HNRNPM	MOG	PSAT1	TNR
ALDH9A1	CKB	GFAP	HNRNPU	MT-CO2	PTBP1	TPI1
ALDOA	CLDN11	GLUD1	HP	MYH9	RACK1	TPP1
ALDOC	CLTC	GLUL	HPX	NACA	RPL37A	TPT1
ANXA2	CNP	GNAO1	HSP90AA1	NCAM1	RPL7A	TRAP1

APOA1	COL1A1	GNB1	HSP90AB1	NCAN	RPL9	TTR
AQP1	COL1A2	GNB4	HSP90B1	NCL	RPLP0	TUBB
AQP4	COX5A	GOT2	HSPA5	NDUFS1	RPS27A	TUBB3
ARF1	COX6A1	GPM6A	HSPA8	NEFM	RPS3A	TUBB4A
ARF3	CRIP2	GSN	HSPA9	NIPSNAP2	RPSA	TUBB6
ARHGDIA	CRMP1	H1-2	HSPB1	NPM1	S100B	TUFM
ATP1A2	CRYAB	H1-3	HSPD1	NSF	SEPTIN2	TXN
ATP2A2	CRYM	H1-4	HSPE1	OXCT1	SERPINA1	TXN2
ATP5F1A	CSRP1	H1-5	IARS1	PA2G4	SET	UBA1
ATP5F1B	CTNNB1	H2AX	IDH3A	PARP1	SH3BGRL	UBE2I
ATP5PB	DHX9	H2AZ1	IGHA1	PCBD1	SLC1A2	USP9X
ATP6V0C	DMWD	H2AZ2	IGHM	PCBP2	SLC1A3	VAPA
ATP6V1A	DPYSL2	H2BC10	IGKC	PDHA1	SLC4A1	VAT1
ATP6V1E1	DPYSL3	H2BC14	IGLC2	PDIA3	SNAP91	VCAN
BCAN	DYNLL1	H2BC15	IGLC3	PEBP1	SOD1	VCP
C1QBP	EEF2	H2BC18	INA	PGAM1	SPTAN1	VDAC2
C3	EIF5A	H2BC5	KHDRBS1	PGK1	SPTBN1	VIM
C4A	ENO1	H2BC9	KRT8	PHB	SSB	VWA5A
C4B	ENO2	H3-3A	LDHA	PHB2	SSBP1	WDR1
CA1	EPB41L2	H3C1	LDHB	PKM	STXBP1	YWHAB
CA2	EPB41L3	H3C13	LMNA	PLEC	SYN1	YWHAZ

Table S2.

The information of antibody

Antibody	Company	Product	Host
HNRNPK	abcam	ab52600	rabbit
β-actin	CST	4970S	Rabbit
CD31	CST	3528S	Mouse
CD44	ProteinTech	60224-1-Ig	Mouse
HIF1A	CST	36169S	Rabbit
OLIG2	CST	65915S	Rabbit
EGFR	CST	4267S	Rabbit
GFAP	ProteinTech	16825-1-AP	Rabbit
PDGFRA	CST	3174S	Rabbit
KI67	Servicebio	GB111499	Rabbit
CREB	ABclonal	A10826	Rabbit
phos-CREB(S133)	ABclonal	AP0019	Rabbit
β-catenin	ProteinTech	51067-2-AP	Rabbit
phos-β-catenin(S37)	ProteinTech	28776-1-AP	Rabbit
GSK-3β	ProteinTech	22104-1-AP	Rabbit
phos-GSK-3β(S9)	Abcam	ab75814	Rabbit
c-JUN	CST	9165S	Rabbit
phos-c-JUN(S73)	CST	3270S	Rabbit
P38	CST	8690T	Rabbit
phos-P38(T180/Y182)	CST	4511S	Rabbit
JNK	CST	9252S	Rabbit

phos-JNK(T183/T185)	CST	4668S	Rabbit
P65	CST	6956S	Mouse

Table S3.

The information of primer

Homo-LGI2-187F	AGGTCTTTGTGGTGGTAGCC
Homo-LGI2-187R	AGACCAGCCTTTGAGCTGTC
Hom-GPNMB-178F	CGGCCAAAGCCATCATAACG
Hom-GPNMB-178R	GAGTTGAGGCCCAAGTGTCA
Homo-TSPAN13-185F	GGGCTGATTTCCAGTCTCCG
Homo-TSPAN13-185R	AGGGCTAAACAAGCGCAAGA
Homo-VPS45-80F	TAACCTGAACCGCACCACTC
Homo-VPS45-80R	CCTCTAGGAAACTTTTCGTGTTGT
Homo-MAGI2-124F	GGGGAAAGCACAAGGGACAT
Homo-MAGI2-124R	GGGCGGGTTCGTCATATTCT
Homo-NFIL3-116F	GGAGCCAAGAGATGACCGAG
Homo-NFIL3-116R	TGGAGGATCGGTTGACTTGC
Homo-UIMC1-132F	GGTATCCTGCCCGCTATGTG
Homo-UIMC1-132R	TCACTCTTGGTCTTGGCCTC
Homo-KAT6A-74F	GCTCCAGTCAGTTCTACACG
Homo-KAT6A-74R	GATGGCTGGCTATTTGCAGG
β-actin-F191	GAAGAGCTACGAGCTGCCTGA
β-actin-R191	CAGACAGCACTGTGTTGGCG

TSPAN13-1-133F	CTCCTGGGGAAACTACACCAA
TSPAN13-1-133R	TGCAAATAAATGCGCTCCGC
TSPAN13-2-137F	GTTCCAAAGCGGGTCCGA
TSPAN13-2-137R	TTCCCTGACAGCTGCTTTGTC

Supplementary Text

molecule sequence

HNRNPK (WT)

ATGGAAACTGAACAGCCAGAAGAAACCTTCCCTAACACTGAAACCAATGGTGAATTTGGTAAAC
GCCCTGCAGAAGATATGGAAGAGGAACAAGCATTTAAAAGATCTAGAAACACTGATGAGATGGT
TGAATTACGCATTCTGCTTCAGAGCAAGAATGCTGGGGCAGTGATTGGAAAAGGAGGCAAGAA
TATTAAGGCTCTCCGTACAGACTACAATGCCAGTGTTTCAGTCCCAGACAGCAGTGGCCCCGA
GCGCATATTGAGTATCAGTGCTGATATTGAAACAATTGGAGAAATTCTGAAGAAAATCATCCCTA
CCTTGGAAGAGGGCCTGCAGTTGCCATCACCCACTGCAACCAGCCAGCTCCCGCTCGAATCTG
ATGCTGTGGAATGCTTAAATTACCAACACTATAAAGGAAGTGACTTTGACTGCGAGTTGAGGCT
GTTGATTCATCAGAGTCTAGCAGGAGGAATTATTGGGGTCAAAGGTGCTAAAATCAAAGAACTT
CGAGAGAACACTCAAACCACCATCAAGCTTTTCCAGGAATGCTGTCCTCATTCCACTGACAGAG
TTGTTCTTATTGGAGGAAAACCCGATAGGGTTGTAGAGTGCATAAAGATCATCCTTGATCTTATA
TCTGAGTCTCCCATCAAAGGACGTGCACAGCCTTATGATCCCAATTTTTACGATGAAACCTATGA
TTATGGTGGTTTTACAATGATGTTTGATGACCGTCGCGGACGCCCAGTGGGATTTCCCATGCGG
GGAAGAGGTGGTTTTGACAGAATGCCTCCTGGTCGGGGTGGGCGTCCCATGCCTCCATCTAGA
AGAGATTATGATGATATGAGCCCTCGTCGAGGACCACCTCCCCCTCCTCCCGGACGAGGCGGC
CGGGGTGGTAGCAGAGCTCGGAATCTTCCTCTTCCTCCACCACCACCACCTAGAGGGGGAGAC
CTCATGGCCTATGACAGAAGAGGGAGACCTGGAGACCGTTACGACGGCATGGTTGGTTTCAGT
GCTGATGAAACTTGGGACTCTGCAATAGATACATGGAGCCCATCAGAATGGCAGATGGCTTATG
AACCACAGGGTGGCTCCGGATATGATTATTCCTATGCAGGGGGTCGTGGCTCATATGGTGATC
TTGGTGGACCTATTATTACTACACAAGTAACTATTCCCAAAGATTTGGCTGGATCTATTATTGGC
AAAGGTGGTCAGCGGATTAAACAAATCCGTCATGAGTCGGGAGCTTCGATCAAAATTGATGAGC
CTTTAGAAGGATCCGAAGATCGGATCATTACCATTACAGGAACACAGGACCAGATACAGAATGC
ACAGTATTTGCTGCAGAACAGTGTGAAGCAGTATGCAGATGTTGAAGGATTCTAA

HNRNPK (K422R)

ATGGAAACTGAACAGCCAGAAGAAACCTTCCCTAACACTGAAACCAATGGTGAATTTGGTAAAC
GCCCTGCAGAAGATATGGAAGAGGAACAAGCATTTAAAAGATCTAGAAACACTGATGAGATGGT
TGAATTACGCATTCTGCTTCAGAGCAAGAATGCTGGGGCAGTGATTGGAAAAGGAGGCAAGAA
TATTAAGGCTCTCCGTACAGACTACAATGCCAGTGTTTCAGTCCCAGACAGCAGTGGCCCCGA
GCGCATATTGAGTATCAGTGCTGATATTGAAACAATTGGAGAAATTCTGAAGAAAATCATCCCTA
CCTTGGAAGAGGGCCTGCAGTTGCCATCACCCACTGCAACCAGCCAGCTCCCGCTCGAATCTG
ATGCTGTGGAATGCTTAAATTACCAACACTATAAAGGAAGTGACTTTGACTGCGAGTTGAGGCT
GTTGATTCATCAGAGTCTAGCAGGAGGAATTATTGGGGTCAAAGGTGCTAAAATCAAAGAACTT
CGAGAGAACACTCAAACCACCATCAAGCTTTTCCAGGAATGCTGTCCTCATTCCACTGACAGAG
TTGTTCTTATTGGAGGAAAACCCGATAGGGTTGTAGAGTGCATAAAGATCATCCTTGATCTTATA
TCTGAGTCTCCCATCAAAGGACGTGCACAGCCTTATGATCCCAATTTTTACGATGAAACCTATGA
TTATGGTGGTTTTACAATGATGTTTGATGACCGTCGCGGACGCCCAGTGGGATTTCCCATGCGG
GGAAGAGGTGGTTTTGACAGAATGCCTCCTGGTCGGGGTGGGCGTCCCATGCCTCCATCTAGA
AGAGATTATGATGATATGAGCCCTCGTCGAGGACCACCTCCCCCTCCTCCCGGACGAGGCGGC
CGGGGTGGTAGCAGAGCTCGGAATCTTCCTCTTCCTCCACCACCACCACCTAGAGGGGGAGAC
CTCATGGCCTATGACAGAAGAGGGAGACCTGGAGACCGTTACGACGGCATGGTTGGTTTCAGT
GCTGATGAAACTTGGGACTCTGCAATAGATACATGGAGCCCATCAGAATGGCAGATGGCTTATG
AACCACAGGGTGGCTCCGGATATGATTATTCCTATGCAGGGGGTCGTGGCTCATATGGTGATC
TTGGTGGACCTATTATTACTACACAAGTAACTATTCCCAAAGATTTGGCTGGATCTATTATTGGC
AAAGGTGGTCAGCGGATTAAACAAATCCGTCATGAGTCGGGAGCTTCGATCAGAATTGATGAG
CCTTTAGAAGGATCCGAAGATCGGATCATTACCATTACAGGAACACAGGACCAGATACAGAATG
CACAGTATTTGCTGCAGAACAGTGTGAAGCAGTATGCAGATGTTGAAGGATTCTAA

KH3 (WT)

ATGATCATCACCACCCAGGTGACCATTCCGAAAGATCTGGCCGGCAGTATTATTGGTAAAGGTG
GCCAGCGTATTAAGCAGATTCGCCATGAAAGTGGTGCCAGTATTAAGATTGATGAACCGCTGGA
AGGTAGTGAAGATCGTATTATTACCATTACCGGTACCCAGGATCAGATTCAGAATGCACAGTAT
CTGCTG

KH3 (K36R)

ATGATCATCACCACCCAGGTTACCATTCCGAAAGATCTGGCCGGCAGTATTATTGGCAAAGGCG
GCCAGCGCATTAAGCAGATTCGTCATGAAAGCGGTGCCAGCATTCGCATTGATGAACCGCTGG

AAGGCAGTGAAGATCGTATTATTACCATTACCGGTACCCAGGATCAGATTCAGAATGCCCAGTA
TCTGCTG

SUMO1

ATGTCTGACCAGGAGGCAAAACCTTCAACTGAGGACTTGGGGGATAAGAAGGAAGGTGAATAT
ATTAAACTCAAAGTCATTGGACAGGATAGCAGTGAGATTCACTTCAAAGTGAAAATGACAACACA
TCTCAAGAAACTCAAAGAATCATACTGTCAAAGACAGGGTGTTCCAATGAATTCACTCAGGTTTC
TCTTTGAGGGTCAGAGAATTGCTGATAATCATACTCCAAAAGAACTGGGAATGGAGGAAGAAGA
TGTGATTGAAGTTTATCAGGAACAAACGGGGGGTCATTCAACAGTTTAG

UBE2I (UBC9)

ATGTCGGGGATCGCCCTCAGCAGACTCGCCCAGGAGAGGAAAGCATGGAGGAAAGACCACCC
ATTTGGTTTCGTGGCTGTCCCAACAAAAAATCCCGATGGCACGATGAACCTCATGAACTGGGAG
TGCGCCATTCCAGGAAAGAAAGGGACTCCGTGGGAAGGAGGCTTGTTTAAACTACGGATGCTT
TTCAAAGATGATTATCCATCTTCGCCACCAAAATGTAAATTCGAACCACCATTATTTCACCCGAA
TGTGTACCCTTCGGGGACAGTGTGCCTGTCCATCTTAGAGGAGGACAAGGACTGGAGGCCAGC
CATCACAATCAAACAGATCCTATTAGGAATACAGGAACTTCTAAATGAACCAAATATCCAAGACC
CAGCTCAAGCAGAGGCCTACACGATTTACTGCCAAAACAGAGTGGAGTACGAGAAAAGGGTCC
GAGCACAAGCCAAGAAGTTTGCGCCCTCATAA

TSPAN13(promoter)

GAGAATAGGGTATCCATCCTCTGAAACATTTATCCTTTGAGTTACAAACAATCCAATTACATTCTT
TAAGTTATTTTAAAATACACAATTAAGTTCTTATTAACGATTGTCACTTTATTGTGCTATCAAATAG
TAGGTCTTATTCATTCTTTCTATTTTTTTGGACCTATTAACCATCCCCACCTTCTCCCGCTCTTGA
TTTTCCTTTGCATGCATGTATTCATAAAGTTTATTAGTATTTTGCAAATCTTTGTAGGATAAGTCT
GACCTGTCCAGGTCACAATTATAGGGCACATATCTTATTTTCTGAGAGAAGCAGGGATTTTATTT
TATTTTTTATTGTTTCACTTATTTTCTCCCTTTCATTTGCCTCTACTTCGTTCTCTTTTAAATCTTTC
ACATGTTTTCATTCATTACTTGCTTCCTGCCTTCCTTCCCTCAAACAAGGCCAGGTGCTTAGTTT
TGAAATCTCATTCAAAATGGTGCCACGTCATCTGCCAGGGCCAAGAATCCAGAGGTGCTGTCAT
ATTTCTCCTTGCCAGCGTGGATCTCCTCCGAGCCCCGCCCTCCCTCCTCACCTGCTCCTGGGG
AAACTACACCAAGGCCGCCGCTCTGGCCTGGGGCTCCCTCCCACACGGCCTTGGCCCTCTCC
CCCTCGCCCCGGGACCGCTCCGCCCCTCCCGGATCCCGGTCGGCGGAGCGCATTTATTTGCA

TATTTCTACCTTTGTTCCCTGCCAGCGGCCAATCAGCGCGGGGCGAGACGAAGGGGCTGG
GCGGGGCTCGGGCTCCTGCT
